# Exocyst-mediated apical Wg secretion activates signaling in the *Drosophila* wing epithelium

**DOI:** 10.1371/journal.pgen.1008351

**Published:** 2019-09-17

**Authors:** Varun Chaudhary, Michael Boutros

**Affiliations:** German Cancer Research Center (DKFZ), Division Signaling and Functional Genomics and Heidelberg University, Department of Cell and Molecular Biology, Im Neuenheimer Feld, Heidelberg, Germany; University of Michigan, UNITED STATES

## Abstract

Wnt proteins are secreted signaling factors that regulate cell fate specification and patterning decisions throughout the animal kingdom. In the *Drosophila* wing epithelium, Wingless (Wg, the homolog of Wnt1) is secreted from a narrow strip of cells at the dorsal-ventral boundary. However, the route of Wg secretion in polarized epithelial cells remains poorly understood and key proteins involved in this process are still unknown. Here, we performed an *in vivo* RNAi screen and identified members of the exocyst complex to be required for apical but not basolateral Wg secretion. Specifically blocking the apical Wg secretion leads to reduced downstream signaling. Using an *in vivo* ‘temporal-rescue’ assay, our results further indicate that apically secreted Wg activates target genes that require high signaling activity. In conclusion, our results demonstrate that the exocyst is required for an apical route of Wg secretion from polarized wing epithelial cells.

## Author summary

Regulation of Wnt signaling and the production of Wnt ligands is crucial for proper development and homeostasis, as dysregulation leads to developmental defects and diseases such as cancer. This study addresses the question of how functional Wnt ligands are secreted by epithelial cells. By using the polarized epithelium of the developing *Drosophila* wing as a model system to study Wnt/Wg secretion, the authors performed a large-scale RNAi screen and identified proteins of the exocyst complex to be required for Wnt signaling. The study shows that exocyst complex preferentially regulates apical secretion of Wg proteins. Taken together, this study identifies routes and regulators for secretion of signaling-active Wnt proteins from polarized epithelial cells.

## Introduction

Wnts are secreted lipid-modified proteins conserved in all animals and play a pivotal role in development and many human diseases such as cancer [1–3]. Initially identified to be important for wing and haltere development in *Drosophila* *[4,5]*, Wnt ligands and their downstream signaling cascades have been subsequently studied in different organisms and many evolutionarily conserved genes required for signaling upstream and downstream of the receptors have been identified [6,7]. Subsequent genetic screens, performed *in vivo* and in cultured *Drosophila* cells, have led to the discovery of components of the Wnt secretion pathway, including the ER membrane protein Porcupine [8], the Wnt cargo receptor Evenness interrupted (Evi; also known as Wntless) [9–11], p24 proteins [12,13] and the retromer complex [14–19]. These factors are required during early steps of Wnt secretion. For example, Evi binds and guides Wnt proteins through the ER and Golgi and loss of Evi leads to block of Wnts in the early secretory compartments [20,21]. In contrast, proteins which may specifically regulate apical or basolateral routes of Wnt secretion in polarized epithelium such as the wing imaginal disc have remained largely unknown.

*Drosophila* wing imaginal discs, which give rise to the adult wing and thorax, are made up of tightly packed columnar epithelial cells (disc proper cells) and a squamous epithelial sheet towards the apical side of disc proper cells called the peripodial membrane [22]. In the wing epithelium, Wg is secreted from two rows of cells at the dorsal-ventral (DV) boundary. Intracellular Wg has been shown to be localized mainly apical [23–25], while extracellular Wg has been reported to be either basolateral [25] or apical [26,27], depending on the method of Wg detection. Recently, it was reported that an E3 ubiquitin ligase called Godzilla mediates apical to basolateral transcytosis of Wg in the producing cells which facilitates signaling from the basolateral side [28]. In contrast, it was also recently reported that Wg is internalized by the receiving cells mainly from the apical surface [29], which raises questions on whether the apical pool of Wg is essential for signaling.

So far, the route of Wg secretion from polarized epithelia has mostly been traced by either following a fluorescently tagged exogenous Wg protein [28,30] or by analyzing the effect of blocking cellular functions like endocytosis on the localization of extracellular Wg [26,27]. These approaches have led to contradictory models of polarized Wg secretion and the route of an untagged endogenous Wg protein remains unknown. Here, we have performed an *in vivo* RNA interference (RNAi) screen to identify regulators of Wg secretion from polarized epithelial cells. The screen identified components of the exocyst complex to be required for polarized Wg secretion. The exocyst complex has been implicated in different cellular trafficking processes, including polarized secretion, ciliogenesis and cell migration [31–33]. Molecularly, the exocyst complex has been described to be required for the fusion of vesicles with a target membrane where it is mostly localized at the site of fusion [31]. We find that the exocyst complex is required for apical secretion of Wg and that specifically blocking the apical route of Wg secretion caused impaired signaling. We further demonstrate that the untagged endogenous Wg is mainly secreted apically and this represents functionally a high-activity pool of Wg proteins.

## Results

### *In vivo* RNAi screening identifies exocyst components as regulators of Wg secretion

**Fig 1:**
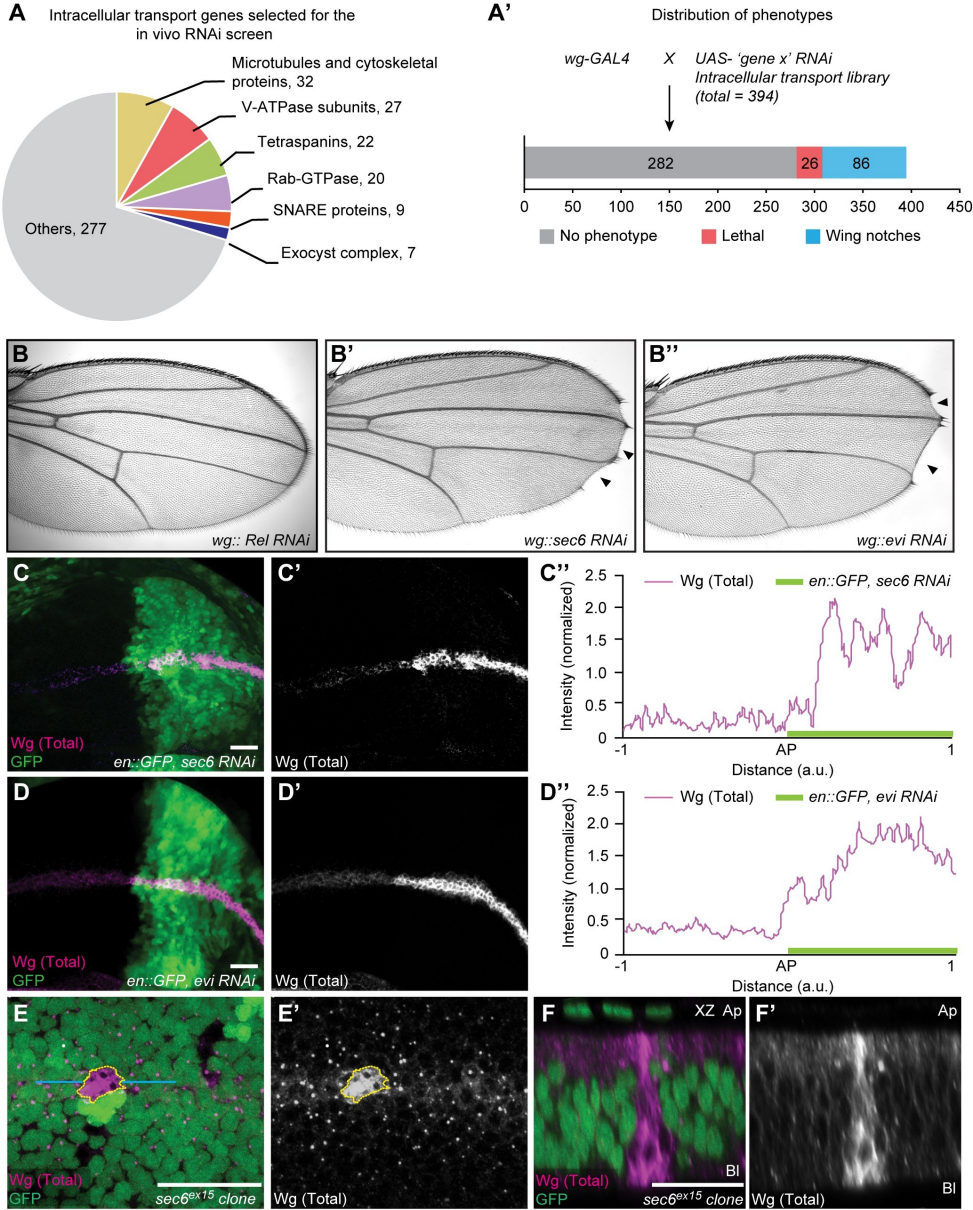
An *in vivo* RNAi screen identifies exocyst components as candidate regulators of Wg secretion. **(A)** A total of 394 genes involved in intracellular transport were selected as input for the *in vivo* screen (see Methods) and some of the functional categories are shown in the pie chart. All the 394 genes were screened *in vivo* using *wg-GAL4* for the expression of RNAi in the Wg producing cells. **(A’)** Distribution of the phenotypes obtained is as follows, 282 no phenotype, 26 lethal (larval/pupal) and 86 wing notch phenotypes. 6 out of 7 screened components of the exocyst complex (marked in black) showed wing notch phenotype upon depletion (see also S1 Table). **(B–B”)** Wing notch phenotype obtained upon depletion of control (gene not related to Wnt signaling) Relish (Rel) (B), Sec6 (B’) and Evi (B”) in Wg producing cells. **(C–D)** Total Wg staining on discs with depletion of Sec6 (C–C”) or Evi (D–D”) in the posterior compartment of the disc (marked by GFP). **(E)** Total Wg accumulation in *sec6*^*ex15*^ mutant clones (marked by absence of GFP). **(F)** XZ section across the *sec6*^*ex15*^ mutant marked by blue lines in E. N≥4 wing discs for C and D and N = 10 for E and F; scale bars 20 μm. a.u. = arbitrary unit. AP = Anterior-Posterior boundary.

We first set out to identify factors involved in the secretion of Wg from polarized cells *in vivo*. We assembled a library of 394 RNAi lines targeting genes with a putative role in protein transport (Fig 1A). Transgenic *UAS* RNAi lines were expressed under the control of *wg-GAL4* to deplete gene products in Wg producing cells. We then scored adult wing notch phenotypes indicative of loss of Wg activity. In total, we identified 86 genes leading to wing notch phenotypes (Fig 1A’ and S1 Table). Among these candidate genes were six (out of eight) components of the exocyst complex (Sec3, Sec5, Sec6, Sec8, Sec15 and Exo84; see S1 Table).

Exocyst proteins form a complex that has an evolutionarily conserved and general role in polarized exocytosis [31]. While its mechanistic functions are not fully understood, it appears to be important for tethering vesicles to the target membrane and for the regulation of SNARE proteins. Here, we used the exocyst complex as a tool to investigate polarized Wg secretion and further focused our experiments on Sec3, Sec6 and Sec15. Validation experiments showed that knockdown of Sec6 in the *wg* expression domain showed a wing notch phenotype similar to the *evi* RNAi control (Fig 1B–1B”). Furthermore, *sec6*, *sec3 and sec15* RNAi in the posterior compartment showed accumulation of total Wg inside the producing cells (Fig 1C–1C”, Fig 1D–1D” and S1A and S1B Fig).

To exclude that *wg* transcription is dependent on Sec6, we tested the expression of a *wg-lacZ* reporter in Sec6 depleted cells. As shown in S1C–S1C” Fig, *wg-lacZ* expression was unaffected by *sec6* RNAi, while Wg accumulation was observed, demonstrating that *wg* transcription is not changed upon Sec6 knockdown. Furthermore, to exclude potential artefacts due to the antibody staining procedures, we assessed whether secretion of GFP-Wg expressed under the control of *Gal4/UAS* system is dependent on Sec6. GFP-Wg protein secretion, as measured by quantification of puncta outside the producing cells, was significantly reduced in *sec6* RNAi, similar to *evi* RNAi positive controls (S1D and S1E Fig). These experiments confirmed that the effect of Sec6 depletion on Wg secretion is neither caused by changes in *wg* transcription nor influenced by the mode of Wg protein detection. Furthermore, we analyzed the phenotype of *sec6* RNAi in a previously described *sec6*^*ex15*^ null allele [34]. *sec6*^*ex15*^ mutant clones showed an intracellular accumulation of Wg (Fig 1E–1E’), which was also visible in transverse sections (Fig 1F–1F’). Taken together, these data suggest that Sec6 and possibly other components of the exocyst complex are required for the secretion of Wg.

### The exocyst is required for apical secretion of Wg

**Fig 2:**
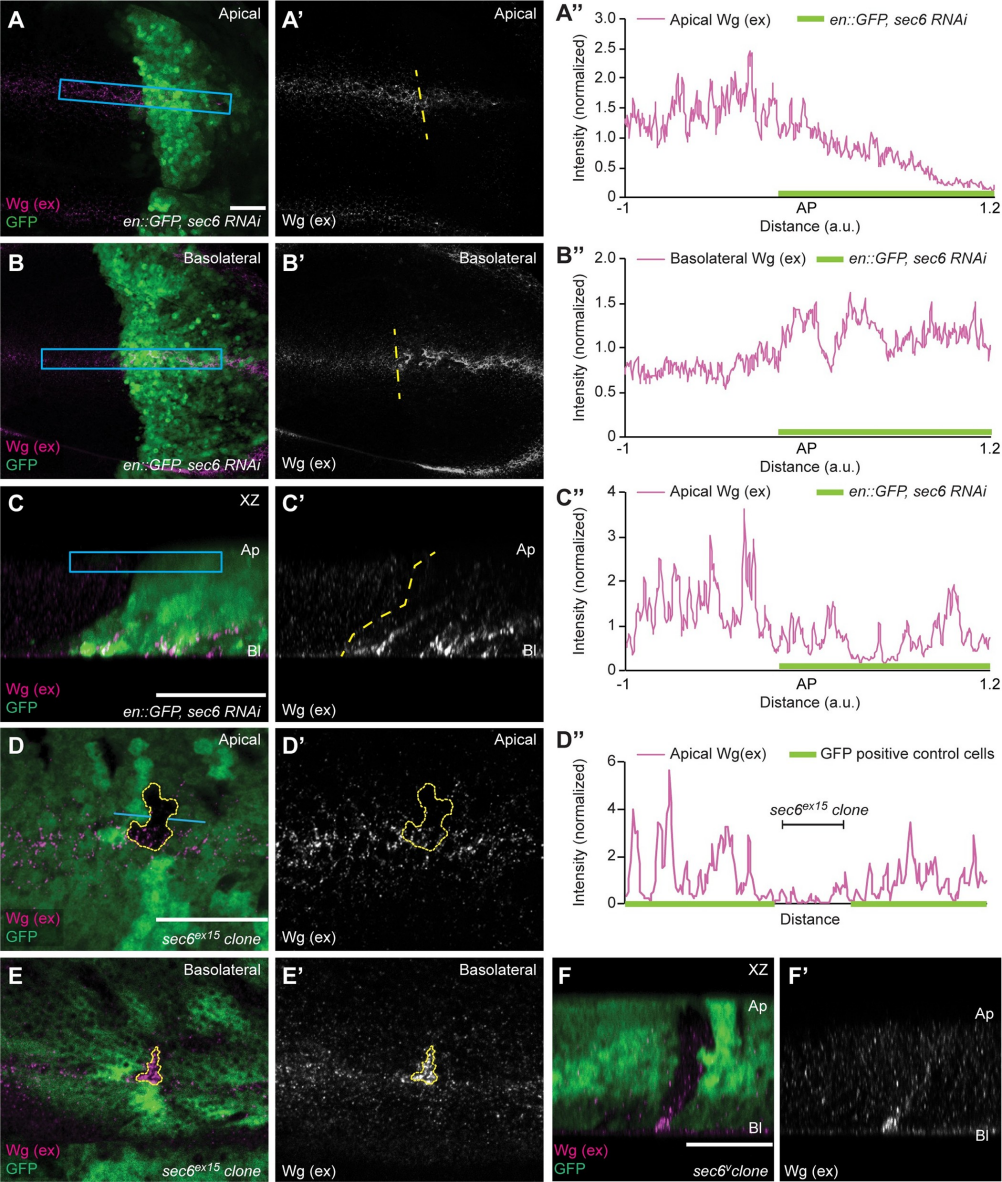
Sec6 depletion blocks apical secretion of Wg. **(A–A”)** Representative images of the apical section of extracellular Wg staining performed on disc with Sec6 depletion in the posterior compartment (marked by GFP). Genotype is *en-Gal4*, *UAS-GFP/UAS-sec6-RNAi*. (A”) Graph shows normalized intensity profile across the blue box in A. **(B–B”)** Images of extracellular Wg staining at the basolateral side of the same Sec6 depleted disc. (B”) Graph shows normalized intensity profile across the blue box in B. **(C–C”)** XZ section of the Sec6 depleted disc shown above. (C”) Normalized intensity profile across the blue box in C. **(D–D”)** Extracellular Wg staining performed on the discs containing small *sec6*^*ex15*^ mutant clones (marked by absence of GFP) shows modest reduction of apical Wg levels, the graph (D”) shows intensity profile of the extracellular Wg across the blue line shown in D. **(E–F)** A clear increase in the basolateral Wg can be seen over the same *sec6*^*ex15*^ mutant clone (E), which is also observed in the XZ section (F). N = 3 discs. Scale bar 20 μm, a.u. = arbitrary unit. AP = Anterior-Posterior boundary.

Next, we investigated the effect of the exocyst in polarized Wg secretion and extracellular Wg distribution in the wing epithelium. To this end, we depleted Sec6 in the posterior compartment of imaginal discs and stained for extracellular Wg. Discs were imaged by confocal sections to visualize Wg protein distribution from the apical to the basolateral side of the epithelium. As shown in Fig 2A–2A”, we observed a significant decrease of extracellular Wg levels at the apical side, as compared to control tissue in the anterior compartment. Furthermore, extracellular Wg levels were increased at the basolateral side (Fig 2B–2B”). Reduced levels of extracellular apical Wg protein were also clearly visible in the transverse sections (Fig 2C–2C’). Quantification of apical Wg (blue box in Fig 2C) showed reduced levels in the Sec6 depleted compartment (Fig 2C”). Moreover, a second, sequence-independent RNAi targeting Sec6 showed similar apical secretion defects (S2A and S2B Fig).

**Fig 3:**
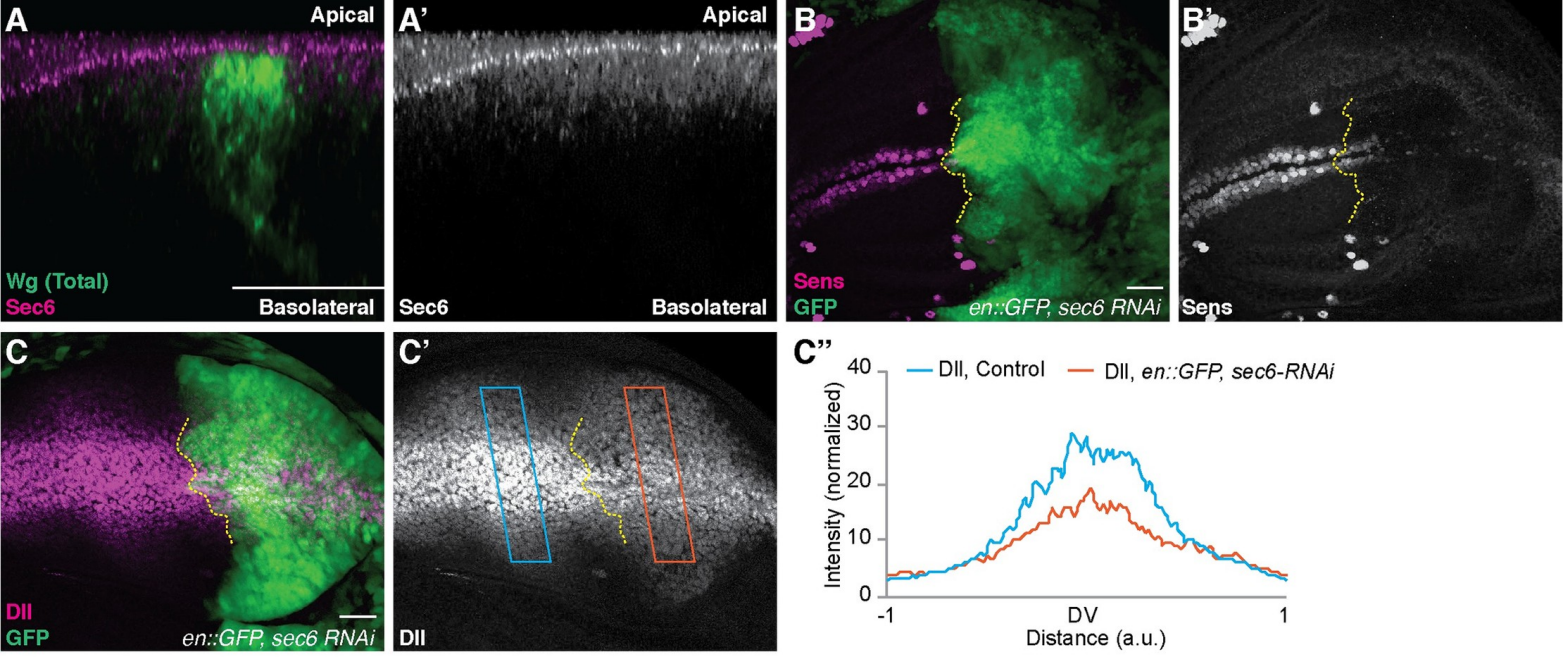
Loss of Sec6 leads to Wg accumulation and loss of canonical signaling. **(A-A’)** Wg and Sec6 antibody staining on the wild-type discs. **(B–B’)** *en-Gal4*, *UAS-GFP/UAS-sec6-RNAi* disc stained for Sens (N = 4). **(C–C”)** *en-Gal4*, *UAS-GFP/ UAS-sec6-RNAi* disc stained for Dll (N = 6). (C”) Quantification of Dll intensity across the blue box (control) and the orange box (Sec6 depleted) shown in C’. Scale bar 20 μm. a.u. = arbitrary unit. DV = Dorsal-Ventral boundary.

We further aimed to confirm these results with a *sec6* mutant allele. While *sec6*^*ex15*^ clones were small, as also previously described [35], which could allow Wg from neighboring unaffected tissue to spread over the mutant clone, in some *sec6*^*ex15*^ clones we observed reduced levels of apical extracellular Wg while basolateral Wg was increased (Fig 2D–2F). We then assessed the localization of Sec6 protein in the wing discs cells by antibody staining. Sec6 was localized on the apical side (Fig 3A–3A’), similar to the previously known apical localization of Sec5 and Sec6 seen in other epithelial cells [35,36]. The localization is consistent with a role of the exocyst complex in mediating exocytosis of Wg at the apical side. Taken together, these experiments showed that Sec6 is required for apical but not basolateral secretion of Wg in wing disc epithelia.

We also analyzed the function of other exocyst complex components in Wg secretion. Extracellular Wg staining on discs with Sec3 knockdown showed reduced apical secretion of Wg (S3A–S3A” Fig) and increased basolateral Wg levels (S3C–S3C” Fig), similar to Sec6 depletion. Expression of *en-Gal4*, *UAS-GFP* was occasionally patchy, leading to mosaic tissue in the posterior compartment with some cells not expressing the transgenes. Consistently, these GFP negative cells showed normal levels of apical extracellular Wg (S3B–S3B’ Fig). In addition, depletion of Sec15 showed reduced apical and increased basolateral Wg levels (S3D and S3E Fig). This further supports our conclusion that the exocyst complex is involved in the apical secretion of Wg.

### Sec6 mediated secretion of Wg is required for high-level of signaling

Wg activates the expression of high-threshold target genes, including the proneural genes *senseless* (*sens)* and *achaete* [37–40] and the low-threshold target gene *Distal-less* (*Dll)* [41–43]. To analyze how loss of Sec6-mediated apical Wg secretion influences downstream signaling, we examined the expression of Sens and Dll upon depletion of Sec6. We observed a loss of Sens expression (Fig 3B–3B’) and a significant reduction of Dll levels (Fig 3C–3C”), indicating a loss of Wg signaling. In order to exclude the possibility that Sec6 is required for Wg signal transduction in the receiving cell, we expressed *sec6* or *evi* RNAi constructs under the control of *apterous-Gal4*. *Apterous (ap)* is expressed in the dorsal compartment of the imaginal disc and its expression overlaps with only one out of two rows of Wg producing cells. Previous studies have demonstrated that Evi is not required in the receiving cells for signal transduction [9,10]. Consistently, we observed that *ap-Gal4>evi RNAi* led to an accumulation of Wg in the dorsal row of producing cells whereas Sens–due to cell non-autonomous rescue by Wg protein secreted from the ventral row of Wg producing cells–remained expressed (S4A and S4B Fig). Similarly, depletion of Sec6 showed retention of Wg only in the dorsal row of producing cells. Sens expression was unaffected on both sides of the DV boundary (S4C Fig). This demonstrates that Sec6 is not required for Wg signal transduction in the receiving cells. We further confirmed these phenotypes by clonal analysis of *sec6*^*ex15*^ mutant alleles. The *sec6*^*ex15*^ clones found in Wg producing cells showed a reduced amount of Sens expression in the nearby Wg receiving cells (S4D and S4D’ Fig, arrows). In contrast, *sec6*^*ex15*^ clones in the Wg receiving cells showed normal expression of Sens (S4E and S4E’ Fig, arrow) and Dll (S4F and S4F’ Fig). Taken together, our data shows that blocking Sec6-mediated apical Wg secretion leads to reduced signaling in the receiving cells, suggesting that the apical pool of extracellular Wg contains high activity.

### Wg is secreted apically from polarized wing epithelial cells

**Fig 4:**
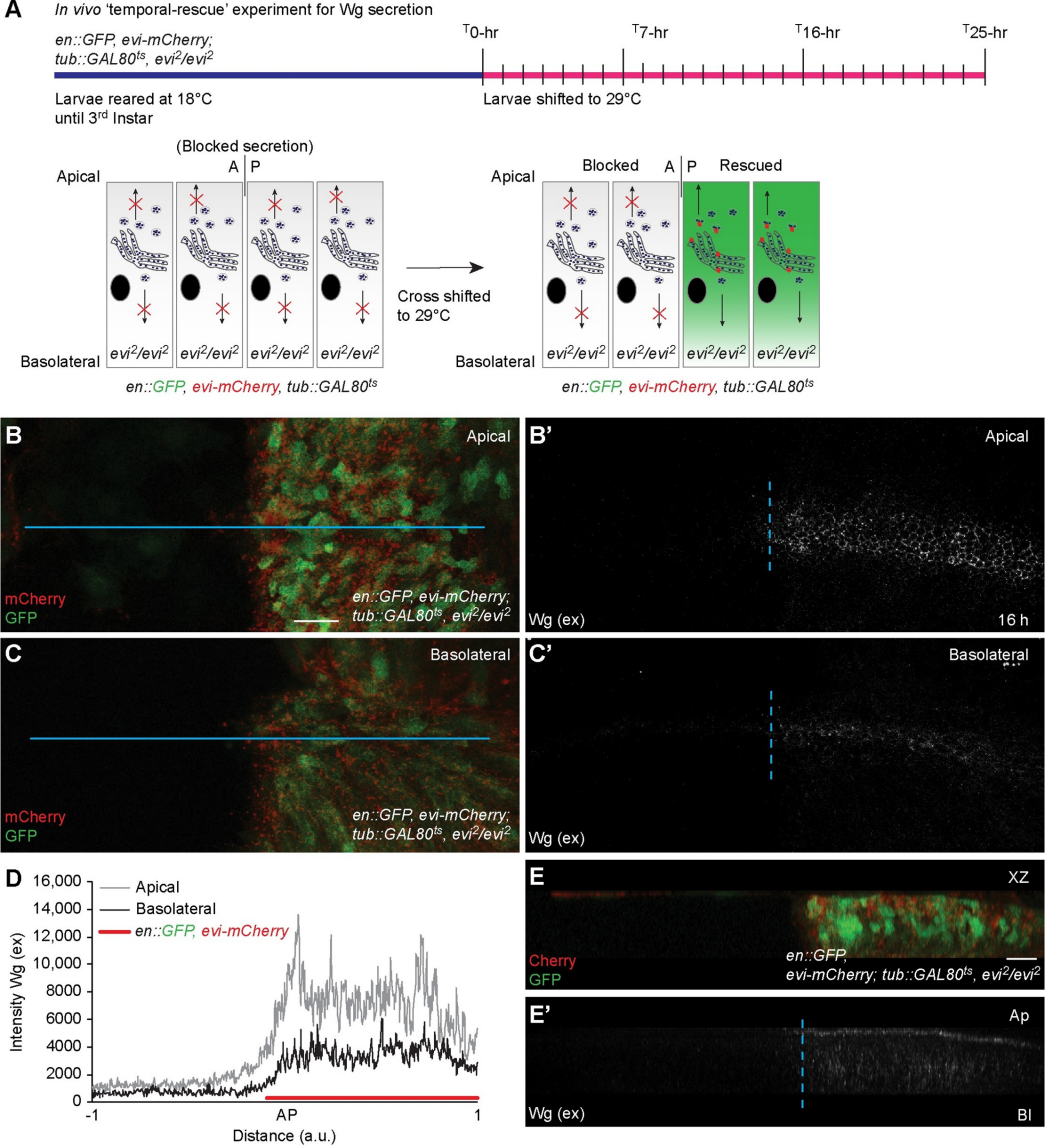
*In vivo* ‘temporal-rescue’ shows Wg secretion from the apical side of the producing cells. **(A)** Scheme for the ‘temporal-rescue’ experiment; *en-Gal4*, *UAS-GFP/UAS-evi-mCherry; tub-Gal80*^*ts*^, *evi*^*2*^*/evi*^*2*^ discs were kept at 18°C until the third-instar stage and then shifted to 29°C for 7h, 16h and 25h. Extracellular Wg staining was performed on discs at these time points. **(B–E)** Representative images of an *evi*^*2*^*/evi*^*2*^ disc rescued by the expression of Evi-mCherry for 16h. Expression of GFP (green) and Evi-mCherry (red) can be observed (B and C). (B’ and C’) shows apical (B’) and basolateral (C’) confocal sections of extracellular Wg staining on the same rescued disc. (D) Quantification of extracellular Wg intensity (across the blue line in B and C). (E and E’) shows XZ view of the disc generated from the confocal stacks. N = 4 discs; Scale bar 20 μm. AP = Anterior-Posterior boundary, a.u. = arbitrary unit.

The polarized secretory route of endogenous Wg from the wing epithelial cells has been controversially discussed and remains largely unclear. To determine the secretion route of untagged endogenous Wg protein, we designed an *in vivo* ‘temporal-rescue’ experiment whereby we block Wg secretion in *evi*^*2*^ homozygous null mutants and then relieve this block by expressing an Evi-mCherry rescue construct under the control of a temperature sensitive *Gal4/Gal80*^*ts*^ expression system (Fig 4A). Using this method, we measured the secretion of Wg protein in a time-resolved manner. Homozygous *evi*^*2*^ mutant larvae expressing GFP and Evi-mCherry specifically in the posterior compartment under the control of *en-Gal4; tub-Gal80*^*ts*^ driver were reared at 18°C (the restrictive temperature for expression) until the third instar stage and then shifted to 29°C (the permissive temperature for expression) for 7, 16 and 25 hours (h) (Fig 4A). Expression of Evi-mCherry was absent at the starting point of the induction (0h) (S6A Fig) and initiated from 7h (S5A Fig and S6B Fig). Concurrently, we detected Wg secretion by extracellular Wg staining from 7h (S5A Fig) with peak secretion at 16h (Fig 4B–4E and S5B Fig), which was also observed until at least 25h (S5C Fig). Analysis of apical and basolateral sections showed higher levels of extracellular Wg present at the apical (Fig 4B–4B’) as compared to the basolateral side of the epithelium (Fig 4C–4C’, Fig 4D and Fig 4E). Taken together these data demonstrate that endogenous Wg is predominantly secreted from the apical surface of the producing cells.

### Apically secreted Wg activates downstream signaling

**Fig 5:**
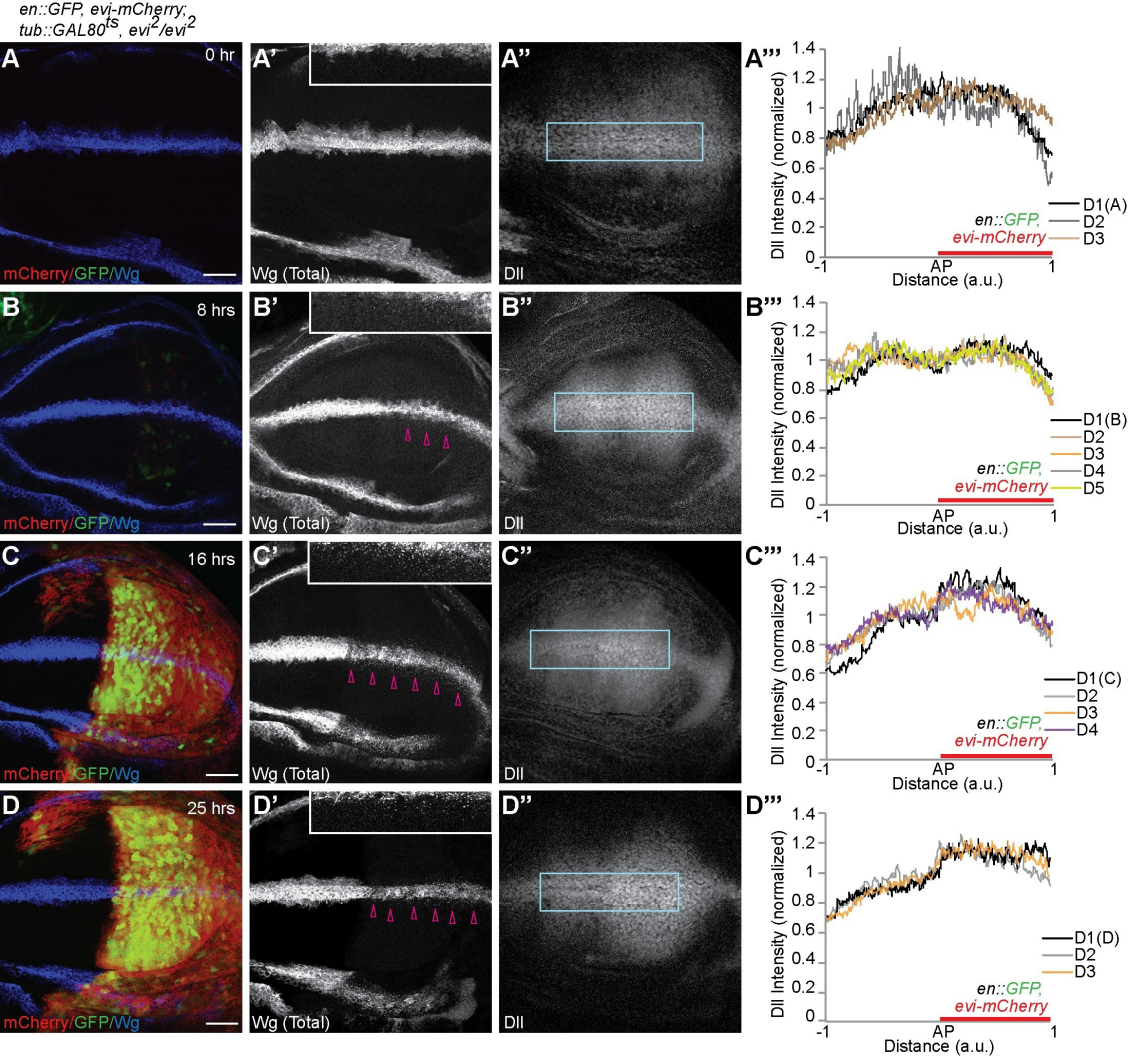
Rescue of Wnt signaling by transient Evi expression. Genotype of all the discs shown in this figure is *en-Gal4*, *UAS-GFP/UAS-evi-mCherry; tub-Gal80*^*ts*^, *evi*^*2*^*/evi*^*2*^. **(A–A”)** Representative images of 0h time point for rescue of *evi*^*2*^*/evi*^*2*^ with Evi-mCherry expression. No expression of GFP or Evi-mCherry can be observed (A). Total Wg staining shows intracellular accumulation of Wg (A’) and Dll expression is unchanged between compartments (A”). Graph (A”’) shows quantification of Dll staining from region marked with blue box in A” (N = 3 discs). **(B–B”)** Representative images of 8h rescue with very weak expression of Evi-mCherry and GFP (B). Total Wg accumulation is mildly reduced (B’, open arrowheads) and Dll is unchanged (B” and also see B”’ for quantification, N = 5 discs). **(C–C”)** Representative images of 16h of rescue showing strong expression of Evi-mCherry and GFP (C), reduced Wg accumulation (C’ open arrowheads) and increased Dll expression (C” and C”’ for quantification, N = 4 discs). **(D–D”)** Total Wg and Dll expression at 25h of rescue (see also D”’ for quantification, N = 3 discs). Insets in A’, B’, C’ and D’ show magnified images of total Wg staining in the posterior compartment. Scale bars 20 μm. AP = Anterior-Posterior boundary, a.u. = arbitrary unit.

Next, to investigate whether the apically secreted Wg can induce signaling in the receiving cells, we analyzed the expression of Wg target genes *sens* and *Dll* in a ‘temporal-rescue’ experiment. We observed that at 0h of Evi-mCherry expression, as expected Wg accumulated in the producing cells (Fig 5A and 5A’). Concurrently, at the 0h time point, Sens expression was not detected (S6A–S6A”” Fig) while low-levels of Dll were found in both anterior and posterior compartments (Fig 5A” and 5A”’), as previously reported to be due to perdurance of maternally provided Evi [9,10] or other ligand-independent mechanisms [44]. After 7-8h of Evi-mCherry expression, when only a small amount of Wg was released (Fig 5B’ open arrowheads and inset), Sens expression remained absent (S6B–S6B”” Fig) and Dll expression was unchanged in the posterior compared to the anterior compartment (Fig 5B” and 5B”’). Subsequently after 16h of Evi-mCherry expression, Sens expression was induced (S6C–S6C”” Fig) and Dll expression increased (Fig 5C” and 5C”’), concurrent with the expression of Evi-mCherry (Fig 5C) and the release of the Wg protein (Fig 5C’ open arrowheads and inset). These results suggest that the strong apical Wg secretion observed at 16h led to the activation of Wg signaling in the receiving cells. Similar results were obtained 25h after the temperature shift (Fig 5D–5D”’ and S6D–S6D”” Fig).

Taken together, these experiments indicate that an apical pool of Wg protein secreted from the DV boundary activates Wnt signaling in the receiving cells, leading to the expression of both high-threshold and low-threshold Wg target genes. Furthermore, these results support the loss of Wg signaling phenotype observed upon blocking apical Wg secretion by Sec6 depletion.

## Discussion

The *Drosophila* wing imaginal disc has been a powerful model to study tissue patterning by secreted signaling molecules, such as Wnt/Wg proteins. Past studies have shown that extracellular Wg protein can be detected at a distance of ten cells or further and this has been proposed to activate the expression of short-range and long-range target genes in a concentration dependent manner [41,45–47]. In contrast, a recent study using membrane-tethered Wg proposed a juxtacrine signaling trafficking of Wg ligands and argued against the requirement of a Wg gradient [48].

While we have learned much about Wnt/Wg secretion in general, the mechanism of polarized Wg secretion has remained unclear. In particular, from which side of the polarized wing epithelial cells the active pool of Wg is secreted has been long debated, and both apical as well as basolateral routes have been suggested. In this study, we demonstrate that a highly active pool of Wg is secreted from the apical side of polarized wing epithelial cells, a process that requires the function of members of the exocyst complex.

### Basolateral and apical mode of Wg secretion in polarized epithelia

At steady-state, extracellular Wg is detected mainly at the basolateral surface of wing epithelial cells [25]. Recently, using a tagged Wg protein expressed via the endogenous Wg enhancer, it was shown that Wg initially reaches the apical surface of the polarized epithelial cells before getting transcytosed to the basolateral side with the help of Godzilla [28]. Loss of Godzilla activity or expression in both Wg producing and receiving cells simultaneously caused an increase of the apical extracellular Wg and loss of signaling. However, as Godzilla is shown to regulate endosomal trafficking in the entire wing pouch region [49], an alternative explanation of the observed results could be that signaling defects observed upon loss of Godzilla are due to its effect on receptor trafficking in the signal receiving cells.

Our experiments provide evidence for the secretion of endogenous untagged Wg from the apical surface. The efficient block of Wg secretion in homozygous *evi* mutants allowed us to perform *in vivo* ‘temporal-rescue’ experiments to visualize Wg secretion in a time-resolved manner, using a staining method to detect extracellular Wg. With these experiments, we found that high amounts of Wg is secreted apically, while only low levels are observed at the basolateral side. This apical secretion observed in our study is also in agreement with previous observations of apical Wg [26] and with earlier studies which show that Wg is internalized from the apical surface of the receiving cells, as visualized by direct endocytic assays [29] or by its apical stabilization upon blocking endocytosis [27]. We further show that apically secreted Wg is functional and is able to activate expression of both low-threshold (*Dll*) and high-threshold (*sens*) target genes in the receiving cells, supporting the importance of this apical Wg pool. However, these experiments do not rule out that a small amount of Wg observed at the basolateral side can also activate low-level signaling.

### The role of the exocyst complex in apical Wg secretion

In an *in vivo* RNAi screen, we first screened for an overall wing margin phenotype. While this phenotype could arise from defects in processes not directly related to Wg secretion, it allowed us to shortlist genes important for wing margin formation. From further analysis, we showed that the exocyst complex is required for apical Wg secretion. The exocyst is believed to act in tethering of secretory vesicles to the plasma membrane and is localized at the sites of exocytosis in several cellular contexts [31]. Consistent with this, we find that Sec6 is localized at the apical side of the wing epithelial cells, supporting a role in regulating fusion of Wg containing vesicles at the apical side. This is also in accordance with its apical localization in polarized pancreatic exocrine cells [50]. Interestingly, the exocyst complex was not identified in cell-based screens for Wnt secretion [10,12,13], which may suggest that it regulates secretion only in polarized cells.

We found that depletion of exocyst complex proteins led to the loss of extracellular Wg at the apical side of the epithelium, with a concomitant increase in basolateral secretion. This basolateral increase in Wg is not observed in the absence of Evi, where Wg remains trapped in the early secretory pathway [20,21], thereby blocking both apical and basolateral Wg secretion. Therefore, our results suggest that Sec6 depletion affects Wg secretion downstream of Evi, specifically blocking vesicle fusion at the apical membrane (our data) [35,36], while allowing the accumulated Wg to be released via the unaffected basolateral route. However, it is also possible that upon Sec6 depletion Wg proteins are channeled more towards the Godzilla-dependent basolateral transcytosis [28]. Nevertheless and more importantly, this exocyst-dependent increase in basolateral Wg did not completely rescue the loss-of apically secreted Wg protein for target gene expression, thus arguing against the basolateral pool of Wg leading to high-level signaling, which is required for the expression of the target gene *sens*. These results also support the previous observation that the apical localization of *wg* transcript is required for proper Wg signaling activity. It was shown that mis-localizing *wg* mRNA to the basolateral side slightly enhanced basolateral distribution, however it led to reduced Wg signaling activity [24].

Moveover, in support to our model, a recent study showed that the exocyst complex regulates apical secretion of Wnt1 in polarized Madin-Darby canine kidney (MDCK) epithelial cells [51], indicating that this function of the exocyst complex might be well-conserved. Our data suggest that the apically secreted Wg, which is difficult to detect at steady-state most likely due to its rapid internalization, is an active pool of Wg. In contrast, the extracellular Wg visible strongly at the basolateral side at steady-state may give an ‘illusion’ of being the only functional pool.

### Mechanisms of polarized Wg signaling

An important question is why apical and basolateral Wg show differential signaling strengths. Several models could explain this differential signaling activities in the polarized wing epithelium: (i) in wing discs, the repertoire of Wnt receptors and co-receptors show polarized localization. A previous study suggested that the basolateral Wg may signal via the Wnt receptor Frizzled2 (Fz2), which is found basolaterally at steady-state [52]. However, Fz2 along with the co-receptor Arrow and Wnt-binding glypican Dally-like are shown to be trafficked and internalized apically [26,27]. Frizzled1, which is redundant with Fz2 for canonical Wnt signaling, is also localized at the apical side [52,53]. (ii) It has been suggested that internalization of Wg is required for proper signaling [27,29,54]. A recent study showed that Wg is internalized from the apical surface of the receiving cells and that these apically derived Wg-containing vesicles fuse with Fz2-containing vesicles inside the cells [29]. Therefore, internalization of Wg from the apical surface of the receiving cells, which will be perturbed upon Sec6 depletion, is needed for its high signaling activity. It is also likely that higher levels of basolateral Wg observed upon depletion of Sec6 is unable to interact with Fz2 in the endosomal compartments and can only activate low-level signaling via Fz2 receptors present at the cell surface. (iii) Different biochemical carriers can influence signaling strengths. Wnt proteins can be secreted on extracellular vesicles [55–59] and bind to extracellular carriers such as lipoprotein particles and Swim [60–62]. This raises the possibility that different signaling abilities are bestowed on Wnt proteins by various carriers and that their different secretion routes in polarized cells can create an activity difference between apical and basolateral Wg.

Taken together, our study provides a mechanism for the secretion of endogenous Wg from polarized epithelial cells and identifies exocyst members required for apical secretion. It will be of further interest to analyze the role of the exocyst in secretion of Wnt proteins in other developmental and niche/stem cell contexts.

## Materials and methods

### *Drosophila* genetics

The following *Drosophila* stocks were used: *wg-GAL4* (2^nd^ chr., a gift from S. Cohen), *ap-GAL4* 2^nd^ chr., [63], *en-GAL4*, *UAS-GFP* 2^nd^ chr. [64], *CyO-wg-LacZ* [65], *UAS-evi-PB-mCherry* *[57]*, *UAS-GFP-wg* [30], *evi*^*2*^ *[10]*, *FRTG13 sec6*^*ex15*^*/CyO-act-GFP* *[34]*, *y*,*w*, *hs-flp; FRTG13 Ubi-GFP M(2)58F/CyO* (a gift from X. Lin), *y*,*w*, *hs-flp; FRTG13 ubi-GFP*.*nls/CyO* (a gift from Y. Bellaiche), *dpp-Gal4/TM6b* (Bloomington stock center stock # 7007), *ubi-GFP FRT2A* (A. Gould). *UAS-sec6-RNAi* (KK-ID 105836 and GD-ID 22077), *UAS-evi-RNAi* (KK-ID 103812), *UAS-sec15-RNAi* (KK-ID 105126), *UAS-sec3-RNAi* (KK-ID 108085), *UAS-Rel-RNAi* (KK-ID 108469) and *UAS-wg-RNAi* (GD-ID 13352) lines were obtained from the Vienna *Drosophila* RNAi Center [66]. All crosses were reared at 25°C except where specifically mentioned.

### *In vivo* RNAi screen

Genes involved in intracellular transport were selected as previously described in [12]. The *in vivo* RNAi screen was performed using the *wg-GAL4* driver to express RNAi transgenes (mostly KK library, some GD library lines, see also S1 Table) in the Wg producing cells. RNAi lines that gave visible wing notches in adult animals were further analyzed. Selected candidates were further studied for defects in Wg secretion by crossing to *en-GAL4*, *UAS-GFP* flies followed by labelling for total and extracellular Wg (data shown only for the components of the exocyst complex). Crosses were reared at 25°C.

### *In vivo* ‘temporal-rescue’ experiments

The *in vivo* ‘temporal-rescue’ experiments of *evi*^*2*^ mutants shown in Figs 4 and 5 and S5 and S6 Figs was performed by keeping larvae of genotype *en-Gal4*, *UAS-GFP/UAS-evi-mCherry; evi*^*2*^, *tub-Gal80*^*ts*^*/evi*^*2*^ at 18°C to prevent Evi expression (permissive temperature for Gal80^ts^) until third instar stage and then shifted to 29°C (restrictive temperature for Gal80^ts^), to induce GFP and Evi-mCherry expression. Larvae were shifted to 29°C for 7, 8, 16 or 25 hours before dissection.

### Heat shock induction of mutant clones

Sec6 loss of function clones were generated by heat shocking larvae of either *y*,*w*, *hs-flp; FRTG13 ubi-nlsGFP/FRTG13 sec6*^*ex15*^ or *y*,*w*, *hs-flp; FRTG13 Ubi-GFP M(2)58F/ FRTG13 sec6*^*ex15*^ genotype, for one hour at 37°C during first- and second-instar larval stages. Wing imaginal discs from wandering third-instar larvae were dissected for antibody staining.

### *Drosophila* genotypes

The following genotypes were used in this study:

Fig 1B: *wg-Gal4/UAS-Rel-RNAi (KK)*

Fig 1B’: *wg-Gal4/UAS-sec6-RNAi (KK)*

Fig 1B”: *wg-Gal4/UAS-evi-RNAi (KK)*

Fig 1C, Fig 2A–2C, Fig 3B and 3C: *en-Gal4*, *UAS-GFP/UAS-sec6-RNAi (KK)*

Fig 1D: *en-Gal4*, *UAS-GFP/UAS-evi-RNAi (KK)*

Fig 1E and 1F, Fig 2D–2F, S4E Fig: *y w hs-FLP/+; FRTG13 sec6*^*ex15*^*/FRTG13 Ubi-nlsGFP*

Fig 3A: *w1118*

Fig 4B–4E, Fig 5A–5D, S5 Fig and S6 Fig: *en-Gal4*, *UAS-GFP/UAS-evi-mCherry; evi*^*2*^, *tub-Gal80*^*ts*^*/evi*^*2*^

S1A Fig, S3A–S3C Fig: *en-Gal4*, *UAS-GFP/UAS-sec3-RNAi (KK)*

S1B Fig, S3D and S3E Fig: *en-Gal4*, *UAS-GFP/UAS-sec15-RNAi (KK)*

S1C Fig: *UAS-sec6-RNAi (KK)/ CyO-wg-LacZ; dpp-Gal4/+*

S1D Fig: *wg-Gal4*, *UAS-GFP-wg/UAS-Rel-RNAi (KK); tub-Gal80*^*ts*^*/+*

S1D’ Fig: *wg-Gal4*, *UAS-GFP-wg/UAS-sec6-RNAi (KK); tub-Gal80*^*ts*^*/+*

S1D” Fig: *wg-Gal4*, *UAS-GFP-wg/UAS-evi-RNAi (KK); tub-Gal80*^*ts*^*/+*

S2A and S2B Fig: *en-Gal4/UAS-sec6-RNAi (GD)*

S4A Fig: *ap-Gal4/+; UAS-GFP/+*

S4B Fig: ap-Gal4/*UAS-evi-RNAi (KK)*; UAS-GFP/+

S4C Fig: ap-Gal4/*UAS-sec6-RNAi (KK)*; UAS-GFP/+

S4D and S4F Fig: *y w hs-FLP/+; FRTG13 sec6*^*ex15*^*/ FRTG13 Ubi-GFP M(2)58F*

### Antibodies

The following antibodies were used: Mouse Anti-Wg (4D4s, obtained from Developmental System Hybridoma Bank) 1:5 for extracellular and 1:50 for total staining, Rabbit-anti-Dll 1:200 (a gift from S. Carroll), Guinea Pig anti-Sens 1:300 [57], Guinea Pig anti-Sec6 1:100 (a gift from U. Tepass). Fluorescent secondary antibodies used were Alexa-405, Alexa-488, Alexa-594 and Alexa-647 (Invitrogen) at 1:300 dilutions.

### Immunostainings, microscopy and image analysis

Extracellular Wg staining was performed as described previously [25]. Wing discs were mounted in Vectashield. Staining and microscopy conditions for discs used for comparisons were identical. Images were taken with a Leica SP5 confocal and were analyzed using ImageJ software [67]. Plot analysis and intensity measurements were performed on raw data processed with ImageJ and statistical analysis was done using Microsoft Excel. Separate channel images were assembled using Adobe Photoshop CS5.1.

### Quantification of GFP-Wg secretion

*wg-GAL4*, *UAS-GFP-wg; tub-Gal80*^*ts*^ flies were crossed to flies containing either *UAS-Rel-RNAi* or *UAS-evi-RNAi* or *UAS-sec6-RNAi*. Crosses were reared at 18°C until early larval stages and were then shifted for 72 hours at 29°C for expression. Wing imaginal discs were imaged using a Leica SP5 confocal and images were generated using ImageJ software [67]. Secreted GFP-Wg punta observed outside the producing cells were separated and quantified by CellProfiler 2.0 software [68].

## Supporting information

S1 Fig Exocyst complex members are required for Wg secretion. **(A–A”)** Depletion of another exocyst component Sec3 in the posterior compartment using *en-Gal4*, *UAS-GFP* shows accumulation of Wg inside the producing cells. **(A”)** The graph shows fold change in the mean intensity between control Wg levels (normalized to 1) and Wg levels in *sec3* RNAi (GFP positive), which are higher than the control (N = 7). **(B–B”)** Depletion of Sec15 in the posterior compartment (GFP positive) of the discs shows accumulation of Wg inside the producing cells. **(C–C”)** *sec6* RNAi was expressed using *dpp-Gal4* (double sided arrows show *dpp* expression domain) in *wg-LacZ* background. No change is observed in the levels of LacZ expression **(C’)** while Wg accumulation can be observed **(C”)**. **(D–E)** GFP-Wg was expressed along with control (gene not related to Wnt signaling) *Relish (Rel)* RNAi *and evi* RNAi or *sec6* RNAi. Quantification shows significant reduction in the secreted GFP-Wg puncta in *sec6* RNAi compared to the control *Rel* RNAi, while *evi* RNAi showed complete loss of GFP-Wg secretion. N = 4 for D and E. error bars s.d.. *P = 0*.*003* generated by Student’s t-test. Scale bar 20 μm.
(TIF)

S2 Fig Sec6 is required for apical Wg secretion. **(A–B)** *en-Gal4*, *UAS-GFP/UAS-sec6 RNAi* (GD) was used to deplete Sec6 in the posterior compartment of the discs (GFP positive). Extracellular Wg staining performed on these discs show reduced apical level **(A–A’)** while the basolateral levels were increased **(B–B’).** Scale bar 20 μm.
(TIF)

S3 Fig Exocyst complex members are required for apical Wg secretion. **(A)** Extracellular Wg staining on disc with Sec3 depletion in the posterior compartment show reduced apical Wg, **(A”)** graph shows normalized mean intensity showing reduced levels of extracellular apical Wg in the RNAi domain (N = 3). **(B–B’)** Enlarged region in A (marked with white box) shows clonal expression of GFP. The GFP negative cells show normal levels of extracellular Wg apically **(B’, Arrow)**. **(C–C”)** The basolateral levels of the extracellular Wg were increased in the *sec3* RNAi expression domain; **(C”)** graph shows normalized mean intensity quantification as above (N = 3). **(D–D”)** Extracellular Wg staining on discs with Sec15 depletion show reduced apical Wg levels. **(E–E”)** While basolateral Wg levels were mildly increased in the posterior *sec15* RNAi compartment **(D”–E”)** Graph shows normalized mean intensity quantification as above (N = 5). Scale bar 20 μm, error bars: s.d.
(TIF)

S4 Fig Sec6 is not required in the receiving cells for transducing Wg signaling. **(A–A”)** Control discs showing expression domain of *ap-Gal4/UAS-GFP* with Sens and Wg staining. **(B–B”)** *evi* RNAi expressed with *ap-GAL4* shows Wg accumulation in one (dorsal) row of producing cells **(B and B”)** and Sens expression is still observed in the dorsal side of the DV boundary **(B’)**. **(C–C”)** Similarly Sec6 depletion shows Wg accumulation in dorsal row of cells while Sens expression is observed. **(D–D’)** Sens staining on disc with *sec6*^*ex15*^ clones (in heterozygous *Minute* background) in the Wg producing cells (yellow dotted line) show reduced Sens expression around the clones (arrows). **(E–E’)** *sec6*^*ex15*^ clones in the Wg receiving cells (yellow dotted line) shows expression of Sens (arrow). **(F–F’)** *sec6*^*ex15*^ clones show normal expression of Dll. Scale bar 20 μm. (N = 3 minimum).
(TIF)

S5 Fig Temporal-Rescue shows apical secretion of Wg. *en-Gal4*, *UAS-GFP/UAS-evi-mCherry; tub-Gal80ts*, *evi*^*2*^*/ evi*^*2*^ discs were kept at 18°C until the third instar stage and then shifted to 29°C for 7 hours (h), 16h and 25h. Extracellular Wg staining was performed on these discs. Panels in **(A)** show 7h expression of GFP (green) and Evi-mCherry (red), where low-level expression of both GFP and Evi-mCherry can be seen (A, left panels). Extracellular Wg staining shows higher levels of Wg at the apical side as compared to the basolateral (A, compare right panels and also see the graph on the right). **(B)** Similarly, panels in B show another example of 16h of expression (as also shown in Fig 4). Strong expression of GFP and Evi-mCherry and higher levels of apical extracellular Wg compared to basolateral Wg were observed. **(C)** Similarly, 25h after expression. Graphs show mean intensity of the extracellular Wg staining across the dotted white line. (N = 3 minimum for each panel), AP = Anterior-Posterior boundary, a.u. = arbitrary unit. Scale bar 20 μm.
(TIF)

S6 Fig Temporal-Rescue of Wg secretion activates sens expression. **(A–A””)** 0 hour of Evi-mCherry expression, where almost no expression of Evi-mCherry (red) and GFP (green) can be observed **(A–A’)** and Wg accumulation in the posterior compartment of the *evi*^*2*^*/evi*^*2*^ discs can still be observed **(A”)**. Sens staining on these discs show no rescue of Sens expression near DV boundary **(A”’–A””)**. **(B–B””)** 7 hours after temperature shift where very weak expression of Evi-mCherry and GFP is observed **(B–B’)**, while total Wg still remained unchanged **(B”)** and Sens expression at the DV boundary remained undetectable **(B”’–B””)**. **(C–C”)** After 16 hours a strong expression of Evi-mCherry and GFP can be observed, moreover Wg accumulation was rescued in the expression domain. **(C”’–C””)**, Weak expression of Sens at the DV boundary (posterior compartment) appears at 16 hours rescue. **(D–D””)** 25 hours of Evi expression shows similar rescue of both Wg accumulation and Sens expression. N≥3 wing discs for each panel; Scale bar 20 μm.
(TIF)

S1 Table *In vivo* RNAi screening results. The above shown genes were selected for i*n vivo* RNAi screen for Wg secretion. All RNAi lines (KK or GD) were crossed with *wg-GAL4 and* crosses were kept at 25°C. Adult wings were scored for notch phenotypes. Six out of eight exocyst complex genes showed notch phenotype (marked in red) and they were identified as a strong candidate for Wg secretion defect. One component (Sec10) was not contained in our library and Exo70 did not give a phenotype.
(PDF)

## References

[1] Logan CY, Nusse R. The Wnt signaling pathway in development and disease. Annu Rev Cell Dev Biol. 2004;20: 781–810. 10.1146/annurev.cellbio.20.010403.113126 15473860

[2] MacDonald BT, Tamai K, He X. Wnt/beta-catenin signaling: components, mechanisms, and diseases. Dev Cell. 2009;17: 9–26. 10.1016/j.devcel.2009.06.016 19619488 PMC2861485

[3] Zhan T, Rindtorff N, Boutros M. Wnt signaling in cancer. Oncogene. 2017;36: 1461–1473. 10.1038/onc.2016.304 27617575 PMC5357762

[4] Sharma RP. Wingless, a new mutant in D. melanogaster. Drosoph Inf Serv. 1973;50: 134.

[5] Sharma RP, Chopra VL. Effect of the wingless (wg1) mutation on wing and haltere development in Drosophila melanogaster. Dev Biol. 1976;48: 461–465. 10.1016/0012-1606(76)90108-1 815114

[6] Niehrs C. The complex world of WNT receptor signalling. Nat Rev Mol Cell Biol. 2012;13: 767–779. 10.1038/nrm3470 23151663

[7] van Amerongen R, Mikels A, Nusse R. Alternative wnt signaling is initiated by distinct receptors. Sci Signal. 2008;1: re9 10.1126/scisignal.135re9 18765832

[8] van den Heuvel M, Harryman-Samos C, Klingensmith J, Perrimon N, Nusse R. Mutations in the segment polarity genes wingless and porcupine impair secretion of the wingless protein. EMBO J. 1993;12: 5293–5302. 8262072 10.1002/j.1460-2075.1993.tb06225.x PMC413795

[9] Bänziger C, Soldini D, Schütt C, Zipperlen P, Hausmann G, Basler K. Wntless, a conserved membrane protein dedicated to the secretion of Wnt proteins from signaling cells. Cell. 2006;125: 509–522. 10.1016/j.cell.2006.02.049 16678095

[10] Bartscherer K, Pelte N, Ingelfinger D, Boutros M. Secretion of Wnt ligands requires Evi, a conserved transmembrane protein. Cell. 2006;125: 523–533. 10.1016/j.cell.2006.04.009 16678096

[11] Goodman RM, Thombre S, Firtina Z, Gray D, Betts D, Roebuck J, et al Sprinter: a novel transmembrane protein required for Wg secretion and signaling. Development. 2006;133: 4901–4911. 10.1242/dev.02674 17108000

[12] Buechling T, Chaudhary V, Spirohn K, Weiss M, Boutros M. p24 proteins are required for secretion of Wnt ligands. EMBO Rep. EMBO Press; 2011;12: 1265–1272. 10.1038/embor.2011.212 PMC3245698 22094269

[13] Port F, Hausmann G, Basler K. A genome-wide RNA interference screen uncovers two p24 proteins as regulators of Wingless secretion. EMBO Rep. 2011;12: 1144–1152. 10.1038/embor.2011.165 21886182 PMC3207098

[14] Coudreuse DYM, Roël G, Betist MC, Destrée O, Korswagen HC. Wnt gradient formation requires retromer function in Wnt-producing cells. Science. 2006;312: 921–924. 10.1126/science.1124856 16645052

[15] Belenkaya TY, Wu Y, Tang X, Zhou B, Cheng L, Sharma YV, et al The retromer complex influences Wnt secretion by recycling wntless from endosomes to the trans-Golgi network. Dev Cell. 2008;14: 120–131. 10.1016/j.devcel.2007.12.003 18160348

[16] Franch-Marro X, Wendler F, Guidato S, Griffith J, Baena-Lopez A, Itasaki N, et al Wingless secretion requires endosome-to-Golgi retrieval of Wntless/Evi/Sprinter by the retromer complex. Nat Cell Biol. 2008;10: 170–177. 10.1038/ncb1678 18193037 PMC7611556

[17] Pan C-L, Baum PD, Gu M, Jorgensen EM, Clark SG, Garriga G. C. elegans AP-2 and retromer control Wnt signaling by regulating mig-14/Wntless. Dev Cell. 2008;14: 132–139. 10.1016/j.devcel.2007.12.001 18160346 PMC2709403

[18] Port F, Kuster M, Herr P, Furger E, Bänziger C, Hausmann G, et al Wingless secretion promotes and requires retromer-dependent cycling of Wntless. Nat Cell Biol. 2008;10: 178–185. 10.1038/ncb1687 18193032

[19] Yang P-T, Lorenowicz MJ, Silhankova M, Coudreuse DYM, Betist MC, Korswagen HC. Wnt signaling requires retromer-dependent recycling of MIG-14/Wntless in Wnt-producing cells. Dev Cell. 2008;14: 140–147. 10.1016/j.devcel.2007.12.004 18160347

[20] Glaeser K, Urban M, Fenech E, Voloshanenko O, Kranz D, Lari F, et al ERAD-dependent control of the Wnt secretory factor Evi. EMBO J. 2018;37 10.15252/embj.201797311 29378775 PMC5813261

[21] Yu J, Chia J, Canning CA, Jones CM, Bard FA, Virshup DM. WLS retrograde transport to the endoplasmic reticulum during Wnt secretion. Dev Cell. 2014;29: 277–291. 10.1016/j.devcel.2014.03.016 24768165

[22] Fristrom DK, Fristrom JW. The metamorphic development of the adult epidermis In: Bate M, A MA, editors. The development of Drosophila melanogaster. Cold Spring Harbor Press; 1993 pp. 843–897.

[23] González F, Swales L, Bejsovec A, Skaer H, Martinez Arias A. Secretion and movement of wingless protein in the epidermis of the Drosophila embryo. Mech Dev. 1991;35: 43–54. 10.1016/0925-4773(91)90040-d 1720017

[24] Simmonds AJ, dosSantos G, Livne-Bar I, Krause HM. Apical Localization of wingless Transcripts Is Required for Wingless Signaling. Cell. 2001;105: 197–207. 10.1016/s0092-8674(01)00311-7 11336670

[25] Strigini M, Cohen SM. Wingless gradient formation in the Drosophila wing. Curr Biol. 2000;10: 293–300. 10.1016/s0960-9822(00)00378-x 10744972

[26] Gallet A, Staccini-Lavenant L, Thérond PP. Cellular trafficking of the glypican Dally-like is required for full-strength Hedgehog signaling and wingless transcytosis. Dev Cell. 2008;14: 712–725. 10.1016/j.devcel.2008.03.001 18477454

[27] Marois E, Mahmoud A, Eaton S. The endocytic pathway and formation of the Wingless morphogen gradient. Development. The Company of Biologists Ltd; 2006;133: 307–317. 10.1242/dev.02197 16354714

[28] Yamazaki Y, Palmer L, Alexandre C, Kakugawa S, Beckett K, Gaugue I, et al Godzilla-dependent transcytosis promotes Wingless signalling in Drosophila wing imaginal discs. Nat Cell Biol. 2016;18: 451–457. 10.1038/ncb3325 26974662 PMC4817240

[29] Hemalatha A, Prabhakara C, Mayor S. Endocytosis of Wingless via a dynamin-independent pathway is necessary for signaling in Drosophila wing discs. Proc Natl Acad Sci U S A. National Academy of Sciences; 2016;113: E6993–E7002. 10.1073/pnas.1610565113 27791132 PMC5111669

[30] Pfeiffer S, Ricardo S, Manneville J-B, Alexandre C, Vincent J-P. Producing Cells Retain and Recycle Wingless in Drosophila Embryos. Curr Biol. 2002;12: 957–962. 10.1016/s0960-9822(02)00867-9 12062063

[31] Heider MR, Munson M. Exorcising the exocyst complex. Traffic. 2012;13: 898–907. 10.1111/j.1600-0854.2012.01353.x 22420621 PMC3374049

[32] Hertzog M, Chavrier P. Cell polarity during motile processes: keeping on track with the exocyst complex. Biochem J. 2011;433: 403–409. 10.1042/BJ20101214 21235523

[33] Liu J, Guo W. The exocyst complex in exocytosis and cell migration. Protoplasma. 2012;249: 587–597. 10.1007/s00709-011-0330-1 21997494

[34] Murthy M, Ranjan R, Denef N, Higashi MEL, Schupbach T, Schwarz TL. Sec6 mutations and the Drosophila exocyst complex. J Cell Sci. 2005;118: 1139–1150. 10.1242/jcs.01644 15728258

[35] Beronja S, Laprise P, Papoulas O, Pellikka M, Sisson J, Tepass U. Essential function of Drosophila Sec6 in apical exocytosis of epithelial photoreceptor cells. J Cell Biol. 2005;169: 635–646. 10.1083/jcb.200410081 15897260 PMC2171699

[36] Murthy M, Teodoro RO, Miller TP, Schwarz TL. Sec5, a member of the exocyst complex, mediates Drosophila embryo cellularization. Development. 2010;137: 2773–2783. 10.1242/dev.048330 20630948 PMC2910387

[37] Phillips RG, Whittle JR. wingless expression mediates determination of peripheral nervous system elements in late stages of Drosophila wing disc development. Development. 1993;118: 427–438. 8223270 10.1242/dev.118.2.427

[38] Nolo R, Abbott LA, Bellen HJ. Senseless, a Zn Finger Transcription Factor, Is Necessary and Sufficient for Sensory Organ Development in Drosophila. Cell. 2000;102: 349–362. 10.1016/s0092-8674(00)00040-4 10975525

[39] Jafar-Nejad H, Tien A-C, Acar M, Bellen HJ. Senseless and Daughterless confer neuronal identity to epithelial cells in the Drosophila wing margin. Development. 2006;133: 1683–1692. 10.1242/dev.02338 16554363

[40] Couso JP, Bishop SA, Martinez Arias A. The wingless signalling pathway and the patterning of the wing margin in Drosophila. Development. 1994;120: 621–636. 8162860 10.1242/dev.120.3.621

[41] Zecca M, Basler K, Struhl G. Direct and Long-Range Action of a Wingless Morphogen Gradient. Cell. 1996;87: 833–844. 10.1016/s0092-8674(00)81991-1 8945511

[42] Carroll SB, Gates J, Keys DN, Paddock SW, Panganiban GE, Selegue JE, et al Pattern formation and eyespot determination in butterfly wings. Science. 1994;265: 109–114. 10.1126/science.7912449 7912449

[43] Diaz-Benjumea FJ, Cohen SM. Serrate signals through Notch to establish a Wingless-dependent organizer at the dorsal/ventral compartment boundary of the Drosophila wing. Development. 1995;121: 4215–4225. 8575321 10.1242/dev.121.12.4215

[44] Chaudhary V, Hingole S, Frei J, Port F, Strutt D, Boutros M. Robust Wnt signaling is maintained by a Wg protein gradient and Fz2 receptor activity in the developing Drosophila wing. Development. 2019;146 10.1242/dev.174789 31399474 PMC6703709

[45] Cadigan KM, Fish MP, Rulifson EJ, Nusse R. Wingless Repression of Drosophila frizzled 2 Expression Shapes the Wingless Morphogen Gradient in the Wing. Cell. 1998;93: 767–777. 10.1016/s0092-8674(00)81438-5 9630221

[46] Neumann CJ, Cohen SM. Long-range action of Wingless organizes the dorsal-ventral axis of the Drosophila wing. Development. 1997;124: 871–880. 9043068 10.1242/dev.124.4.871

[47] Struhl G, Basler K. Organizing activity of wingless protein in Drosophila. Cell. 1993;72: 527–540. 10.1016/0092-8674(93)90072-x 8440019

[48] Alexandre C, Baena-Lopez A, Vincent J-P. Patterning and growth control by membrane-tethered Wingless. Nature. 2014;505: 180–185. 10.1038/nature12879 24390349 PMC7611559

[49] Yamazaki Y, Schönherr C, Varshney GK, Dogru M, Hallberg B, Palmer RH. Goliath family E3 ligases regulate the recycling endosome pathway via VAMP3 ubiquitylation. EMBO J. 2013;32: 524–537. 10.1038/emboj.2013.1 23353890 PMC3579141

[50] Shin DM, Zhao XS, Zeng W, Mozhayeva M, Muallem S. The mammalian Sec6/8 complex interacts with Ca(2+) signaling complexes and regulates their activity. J Cell Biol. 2000;150: 1101–1112. 10.1083/jcb.150.5.1101 10973998 PMC2175249

[51] Yamamoto H, Sato A, Kikuchi A. Apical secretion of Wnt1 in polarized epithelial cells is regulated by exocyst-mediated trafficking. J Biochem. 2017;162: 317–326. 10.1093/jb/mvx035 28992081

[52] Wu J, Klein TJ, Mlodzik M. Subcellular localization of frizzled receptors, mediated by their cytoplasmic tails, regulates signaling pathway specificity. PLoS Biol. 2004;2: E158 10.1371/journal.pbio.0020158 15252441 PMC449784

[53] Strutt DI. Asymmetric Localization of Frizzled and the Establishment of Cell Polarity in the Drosophila Wing. Mol Cell. 2001;7: 367–375. 11239465 10.1016/s1097-2765(01)00184-8

[54] Seto ES, Bellen HJ. Internalization is required for proper Wingless signaling in Drosophila melanogaster. J Cell Biol. 2006;173: 95–106. 10.1083/jcb.200510123 16606693 PMC2063794

[55] Beckett K, Monier S, Palmer L, Alexandre C, Green H, Bonneil E, et al Drosophila S2 cells secrete wingless on exosome-like vesicles but the wingless gradient forms independently of exosomes. Traffic. 2013;14: 82–96. 10.1111/tra.12016 23035643 PMC4337976

[56] Greco V, Hannus M, Eaton S. Argosomes: a potential vehicle for the spread of morphogens through epithelia. Cell. 2001;106: 633–645. 10.1016/s0092-8674(01)00484-6 11551510

[57] Gross JC, Chaudhary V, Bartscherer K, Boutros M. Active Wnt proteins are secreted on exosomes. Nat Cell Biol. 2012;14: 1036–1045. 10.1038/ncb2574 22983114

[58] Korkut C, Ataman B, Ramachandran P, Ashley J, Barria R, Gherbesi N, et al Trans-synaptic transmission of vesicular Wnt signals through Evi/Wntless. Cell. 2009;139: 393–404. 10.1016/j.cell.2009.07.051 19837038 PMC2785045

[59] Luga V, Zhang L, Viloria-Petit AM, Ogunjimi AA, Inanlou MR, Chiu E, et al Exosomes mediate stromal mobilization of autocrine Wnt-PCP signaling in breast cancer cell migration. Cell. 2012;151: 1542–1556. 10.1016/j.cell.2012.11.024 23260141

[60] Neumann S, Coudreuse DYM, van der Westhuyzen DR, Eckhardt ERM, Korswagen HC, Schmitz G, et al Mammalian Wnt3a is released on lipoprotein particles. Traffic. 2009;10: 334–343. 10.1111/j.1600-0854.2008.00872.x 19207483

[61] Panáková D, Sprong H, Marois E, Thiele C, Eaton S. Lipoprotein particles are required for Hedgehog and Wingless signalling. Nature. 2005;435: 58–65. 10.1038/nature03504 15875013

[62] Mulligan KA, Fuerer C, Ching W, Fish M, Willert K, Nusse R. Secreted Wingless-interacting molecule (Swim) promotes long-range signaling by maintaining Wingless solubility. Proc Natl Acad Sci U S A. 2012;109: 370–377. 10.1073/pnas.1119197109 22203956 PMC3258625

[63] Montagne J, Stewart MJ, Stocker H, Hafen E, Kozma SC, Thomas G. Drosophila S6 kinase: a regulator of cell size. Science. 1999;285: 2126–2129. 10.1126/science.285.5436.2126 10497130

[64] Thompson BJ, Cohen SM. The Hippo pathway regulates the bantam microRNA to control cell proliferation and apoptosis in Drosophila. Cell. 2006;126: 767–774. 10.1016/j.cell.2006.07.013 16923395

[65] Kassis JA, Noll E, VanSickle EP, Odenwald WF, Perrimon N. Altering the insertional specificity of a Drosophila transposable element. Proc Natl Acad Sci U S A. National Academy of Sciences; 1992;89: 1919–1923. 10.1073/pnas.89.5.1919 1311855 PMC48565

[66] Dietzl G, Chen D, Schnorrer F, Su K-C, Barinova Y, Fellner M, et al A genome-wide transgenic RNAi library for conditional gene inactivation in Drosophila. Nature. 2007;448: 151–156. 10.1038/nature05954 17625558

[67] Schneider CA, Rasband WS, Eliceiri KW. NIH Image to ImageJ: 25 years of image analysis. Nat Methods. 2012;9: 671–675. 10.1038/nmeth.2089 22930834 PMC5554542

[68] Carpenter AE, Jones TR, Lamprecht MR, Clarke C, Kang IH, Friman O, et al CellProfiler: image analysis software for identifying and quantifying cell phenotypes. Genome Biol. 2006;7: R100 10.1186/gb-2006-7-10-r100 17076895 PMC1794559

