# Supplementary figures and images for: Exocyst-mediated apical Wg secretion activates signaling in the Drosophila wing epithelium
Source: PLoS Genet. 2019 Sep 17;15(9):e1008351. doi: 10.1371/journal.pgen.1008351 (PMC6764796; doi:10.1371/journal.pgen.1008351)

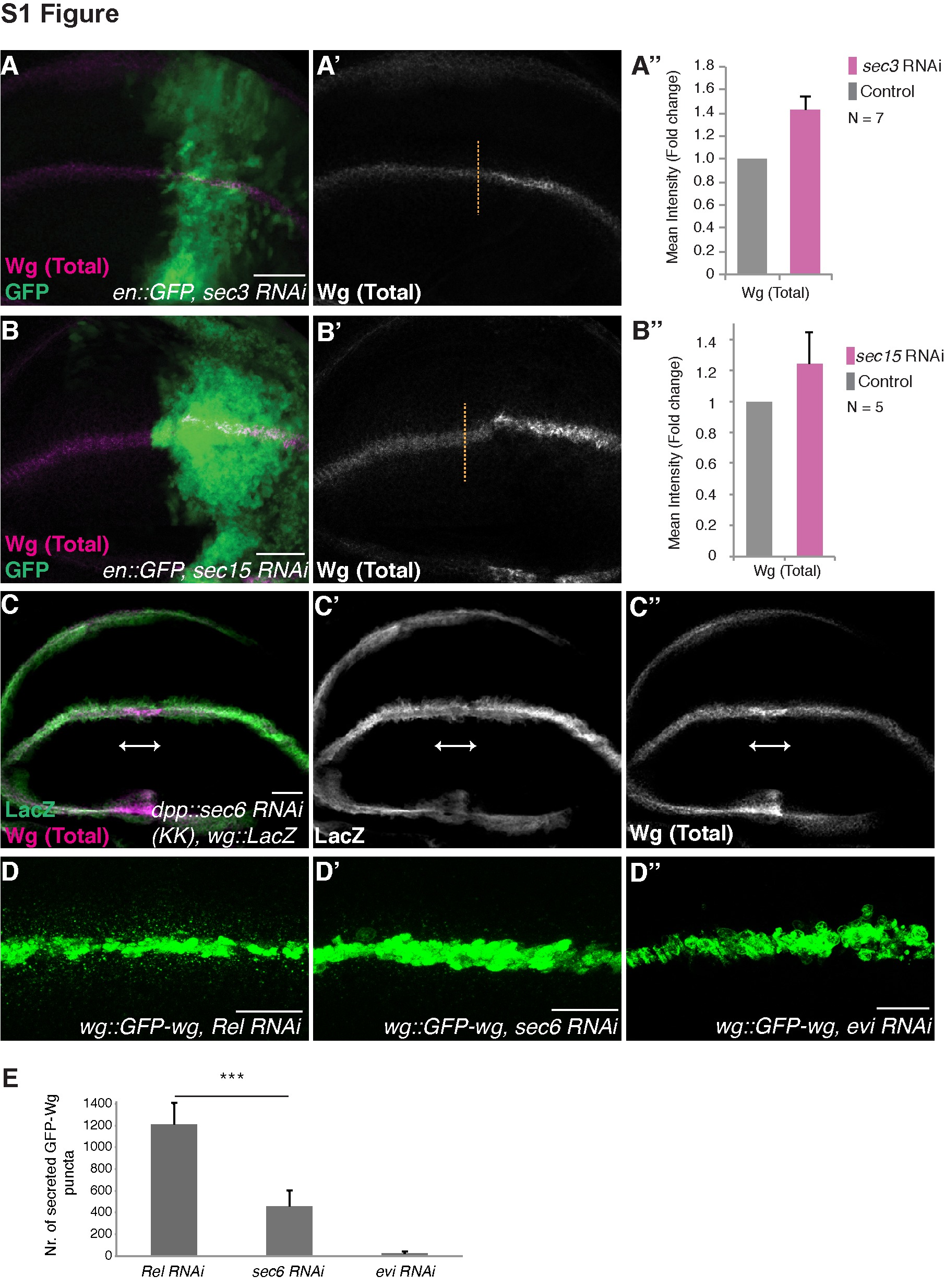

Supplement: S1 Fig — (A–A”) Depletion of another exocyst component Sec3 in the posterior compartment using en-Gal4, UAS-GFP shows accumulation of Wg inside the producing cells. (A”) The graph shows fold change in the mean intensity between control Wg levels (normalized to 1) and Wg levels in sec3 RNAi (GFP positive), which are higher than the control (N = 7). (B–B”) Depletion of Sec15 in the posterior compartment (GFP positive) of the discs shows accumulation of Wg inside the producing cells. (C–C”) sec6 RNAi was expressed using dpp-Gal4 (double sided arrows show dpp expression domain) in wg-LacZ background. No change is observed in the levels of LacZ expression (C’) while Wg accumulation can be observed (C”). (D–E) GFP-Wg was expressed along with control (gene not related to Wnt signaling) Relish (Rel) RNAi and evi RNAi or sec6 RNAi. Quantification shows significant reduction in the secreted GFP-Wg puncta in sec6 RNAi compared to the control Rel RNAi, while evi RNAi showed complete loss of GFP-Wg secretion. N = 4 for D and E. error bars s.d.. P = 0.003 generated by Student’s t-test. Scale bar 20 μm. (TIF) [file pgen.1008351.s001.tif]

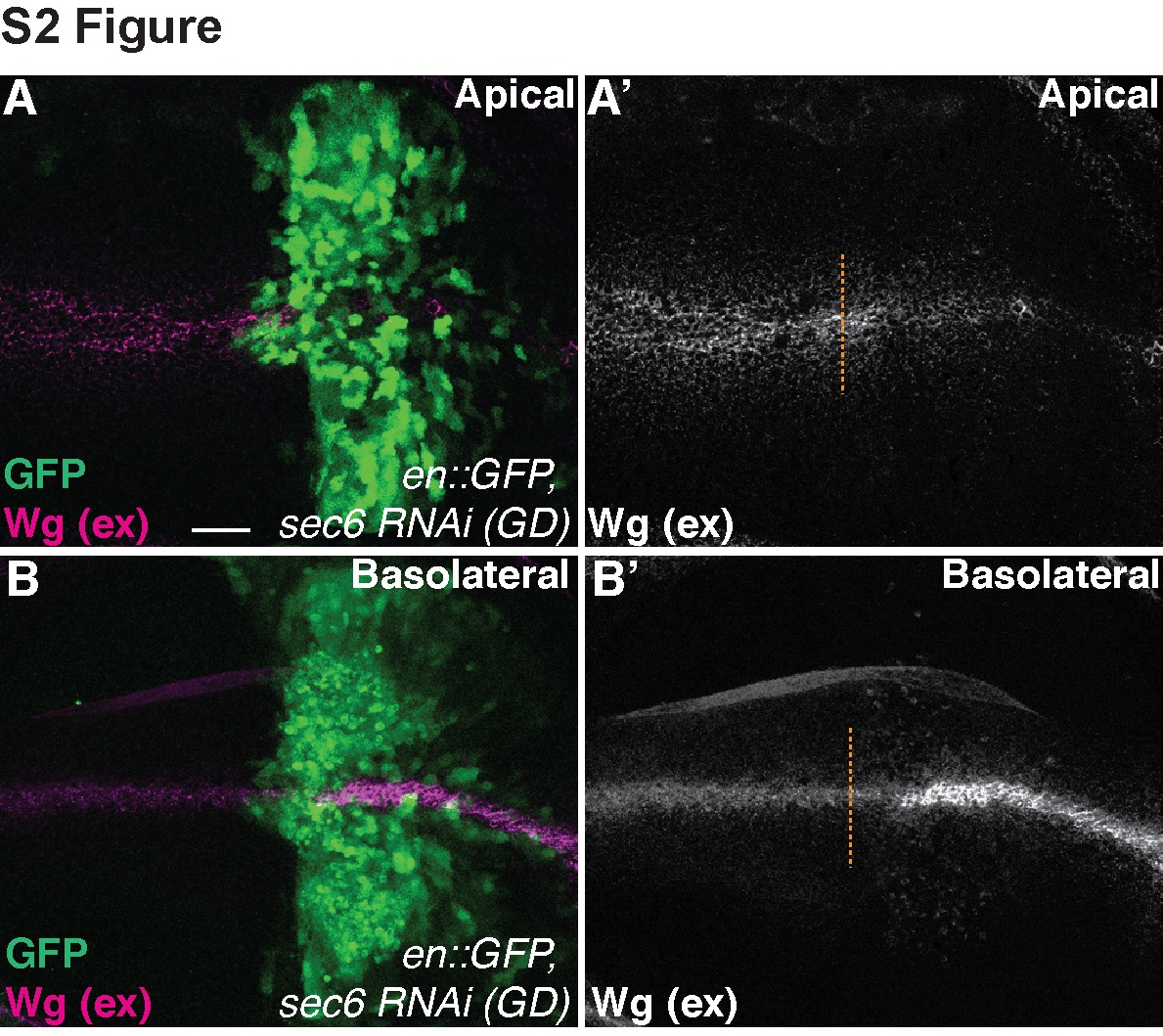

Supplement: S2 Fig — (A–B) en-Gal4, UAS-GFP/UAS-sec6 RNAi (GD) was used to deplete Sec6 in the posterior compartment of the discs (GFP positive). Extracellular Wg staining performed on these discs show reduced apical level (A–A’) while the basolateral levels were increased (B–B’). Scale bar 20 μm. (TIF) [file pgen.1008351.s002.tif]

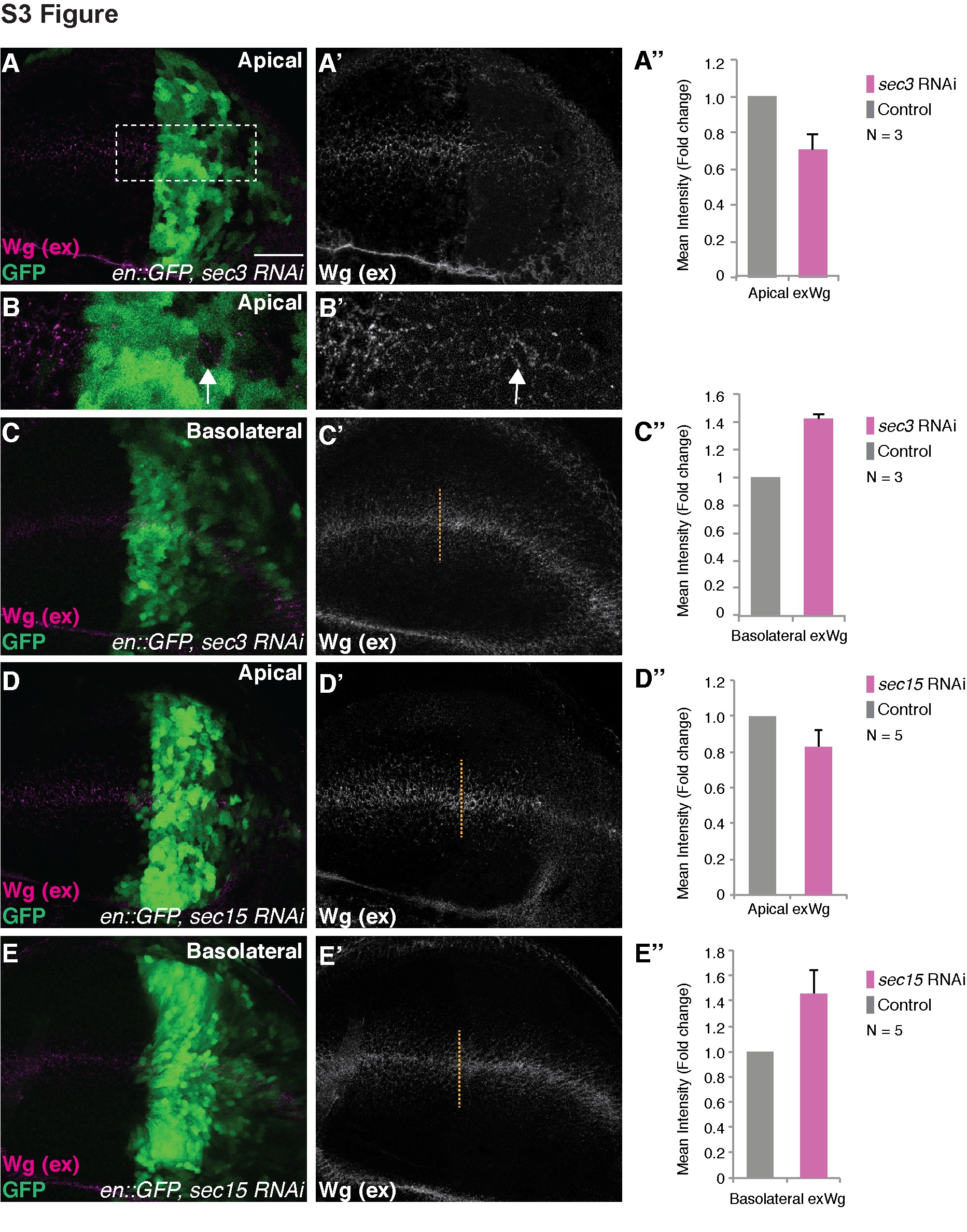

Supplement: S3 Fig — (A) Extracellular Wg staining on disc with Sec3 depletion in the posterior compartment show reduced apical Wg, (A”) graph shows normalized mean intensity showing reduced levels of extracellular apical Wg in the RNAi domain (N = 3). (B–B’) Enlarged region in A (marked with white box) shows clonal expression of GFP. The GFP negative cells show normal levels of extracellular Wg apically (B’, Arrow). (C–C”) The basolateral levels of the extracellular Wg were increased in the sec3 RNAi expression domain; (C”) graph shows normalized mean intensity quantification as above (N = 3). (D–D”) Extracellular Wg staining on discs with Sec15 depletion show reduced apical Wg levels. (E–E”) While basolateral Wg levels were mildly increased in the posterior sec15 RNAi compartment (D”–E”) Graph shows normalized mean intensity quantification as above (N = 5). Scale bar 20 μm, error bars: s.d. (TIF) [file pgen.1008351.s003.tif]

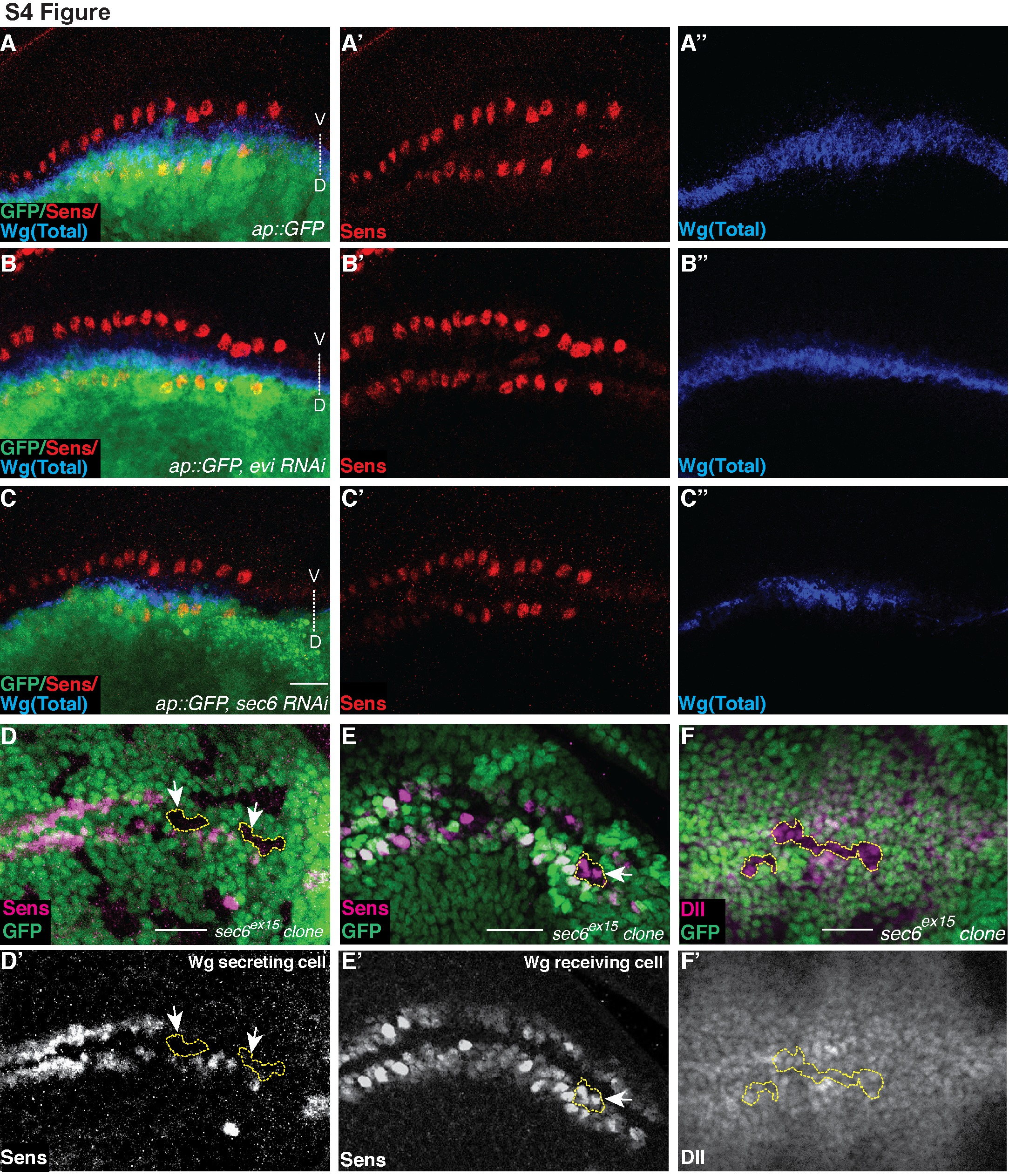

Supplement: S4 Fig — (A–A”) Control discs showing expression domain of ap-Gal4/UAS-GFP with Sens and Wg staining. (B–B”) evi RNAi expressed with ap-GAL4 shows Wg accumulation in one (dorsal) row of producing cells (B and B”) and Sens expression is still observed in the dorsal side of the DV boundary (B’). (C–C”) Similarly Sec6 depletion shows Wg accumulation in dorsal row of cells while Sens expression is observed. (D–D’) Sens staining on disc with sec6ex15 clones (in heterozygous Minute background) in the Wg producing cells (yellow dotted line) show reduced Sens expression around the clones (arrows). (E–E’) sec6ex15 clones in the Wg receiving cells (yellow dotted line) shows expression of Sens (arrow). (F–F’) sec6ex15 clones show normal expression of Dll. Scale bar 20 μm. (N = 3 minimum). (TIF) [file pgen.1008351.s004.tif]

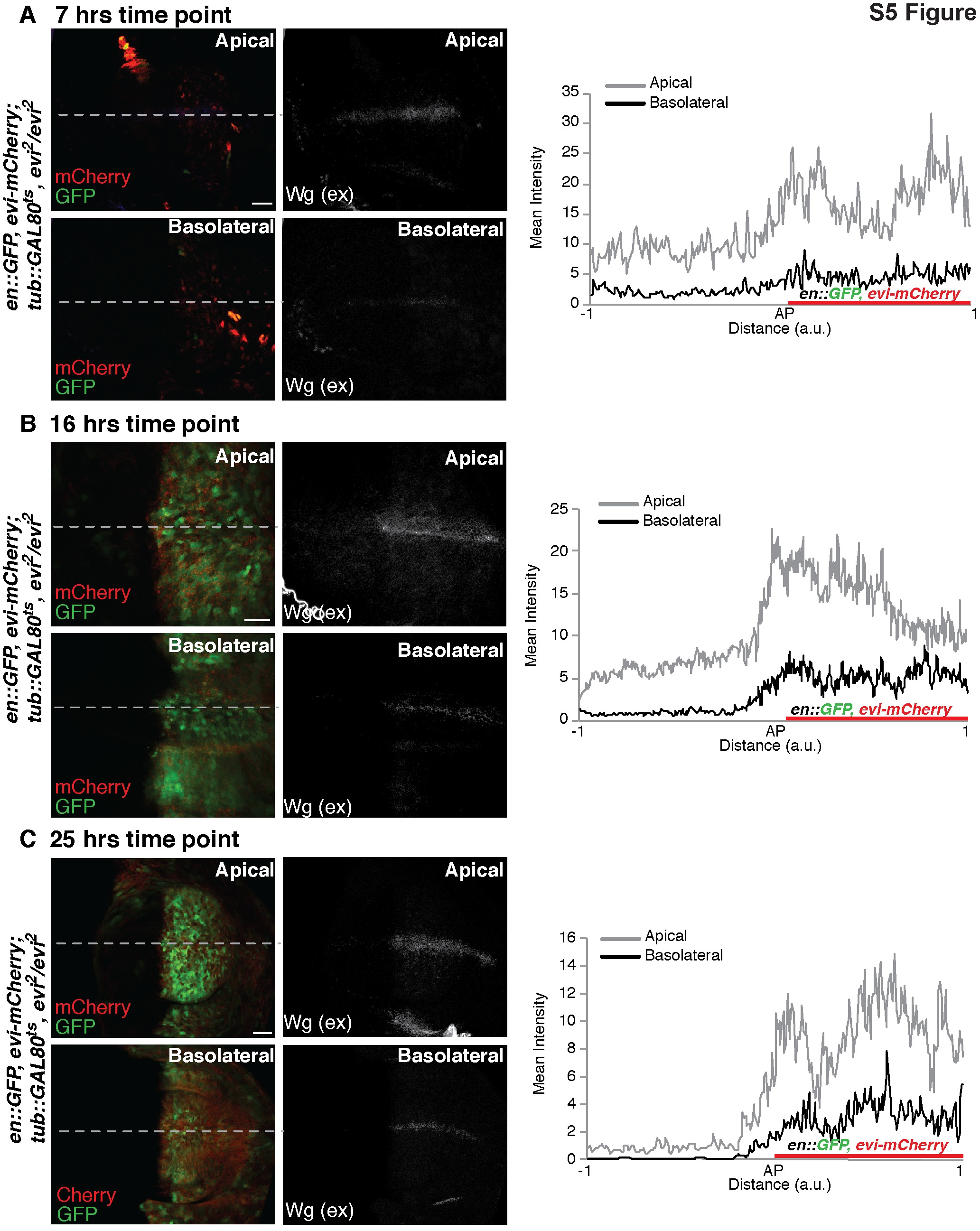

Supplement: S5 Fig — en-Gal4, UAS-GFP/UAS-evi-mCherry; tub-Gal80ts, evi2/ evi2 discs were kept at 18°C until the third instar stage and then shifted to 29°C for 7 hours (h), 16h and 25h. Extracellular Wg staining was performed on these discs. Panels in (A) show 7h expression of GFP (green) and Evi-mCherry (red), where low-level expression of both GFP and Evi-mCherry can be seen (A, left panels). Extracellular Wg staining shows higher levels of Wg at the apical side as compared to the basolateral (A, compare right panels and also see the graph on the right). (B) Similarly, panels in B show another example of 16h of expression (as also shown in Fig 4). Strong expression of GFP and Evi-mCherry and higher levels of apical extracellular Wg compared to basolateral Wg were observed. (C) Similarly, 25h after expression. Graphs show mean intensity of the extracellular Wg staining across the dotted white line. (N = 3 minimum for each panel), AP = Anterior-Posterior boundary, a.u. = arbitrary unit. Scale bar 20 μm. (TIF) [file pgen.1008351.s005.tif]

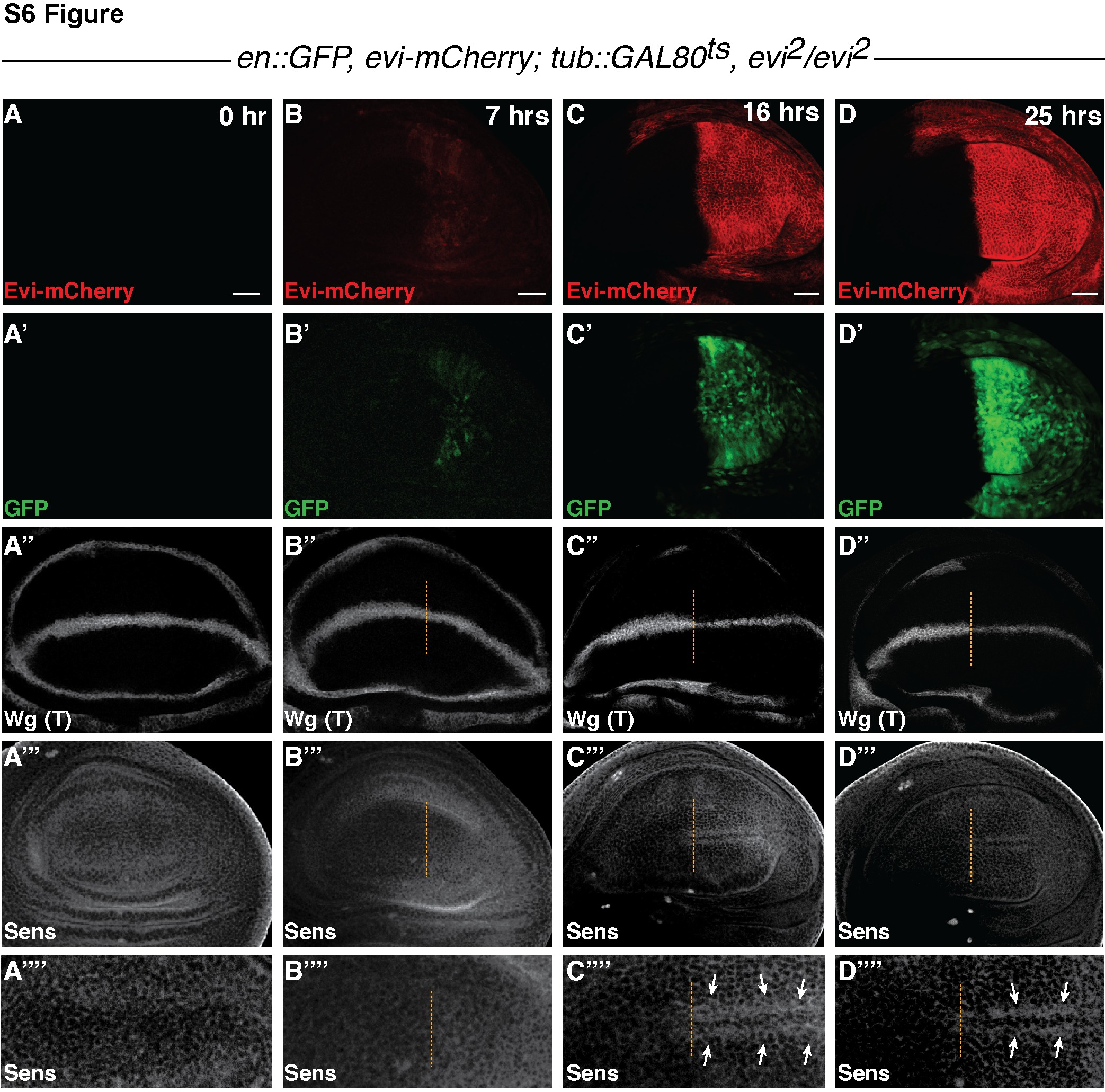

Supplement: S6 Fig — (A–A””) 0 hour of Evi-mCherry expression, where almost no expression of Evi-mCherry (red) and GFP (green) can be observed (A–A’) and Wg accumulation in the posterior compartment of the evi2/evi2 discs can still be observed (A”). Sens staining on these discs show no rescue of Sens expression near DV boundary (A”’–A””). (B–B””) 7 hours after temperature shift where very weak expression of Evi-mCherry and GFP is observed (B–B’), while total Wg still remained unchanged (B”) and Sens expression at the DV boundary remained undetectable (B”’–B””). (C–C”) After 16 hours a strong expression of Evi-mCherry and GFP can be observed, moreover Wg accumulation was rescued in the expression domain. (C”’–C””), Weak expression of Sens at the DV boundary (posterior compartment) appears at 16 hours rescue. (D–D””) 25 hours of Evi expression shows similar rescue of both Wg accumulation and Sens expression. N≥3 wing discs for each panel; Scale bar 20 μm. (TIF) [file pgen.1008351.s006.tif]
