# Supplementary material for: Exocyst-mediated apical Wg secretion activates signaling in the Drosophila wing epithelium
Source: PLoS Genet. 2019 Sep 17;15(9):e1008351. doi: 10.1371/journal.pgen.1008351 (PMC6764796; doi:10.1371/journal.pgen.1008351)
Supplement: S1 Table — The above shown genes were selected for in vivo RNAi screen for Wg secretion. All RNAi lines (KK or GD) were crossed with wg-GAL4 and crosses were kept at 25°C. Adult wings were scored for notch phenotypes. Six out of eight exocyst complex genes showed notch phenotype (marked in red) and they were identified as a strong candidate for Wg secretion defect. One component (Sec10) was not contained in our library and Exo70 did not give a phenotype. (PDF) [file pgen.1008351.s007.pdf]

Table S1: *In vivo* RNAi screening results; Related to Figure 1

Nr. of genes	Nr. of RNAi lines	AnnotationID	Current ID	Gene symbol	Gene Name	VDRC RNAi Library	Transformant ID	*wg-GAL4* cross Adult wing phenotype	Remarks
1	1	CG17515	FBgn0039966	Rab21	Rab21	GD	32940	Lethal/Notches	
**2**	**2**	**CG6095**	**FBgn0260946**	**exo84**	**exo84**	**KK**	**108650**	**Lethal/Notches**	
3	3	CG13281	FBgn0022213	Cas	CAS/CSE1 segregation protein	KK	110215	Lethal/Notches	
4	4	CG1938	FBgn0030276	Dlic	Dynein light intermediate chain	KK	101340	Lethal/Notches	
5	5	CG8532	FBgn0028582	lqf	liquid facets	KK	107300	Lethal/Notches	
6	6	CG3664	FBgn0014010	Rab5	Rab-protein 5	KK	103945	Lethal (early)	
7	7	CG8210	FBgn0262512	Vha14-1	Vacuolar H+ ATPase 14kD subunit	KK	110160	Lethal	
8	8	CG3762	FBgn0263598	Vha68-2	Vha68-2	KK	110600	Lethal	
9	9	CG10130	FBgn0010638	Sec61beta	Sec61beta	KK	107784	Lethal	
10	10	CG6842	FBgn0027605	Vps4	bcDNA:GH02678,BcDNA:...	GD	35126	Lethal	
11	11	CG4071	FBgn0034744	Vps20	anon-EST:Posey40,vps...	KK	103944	Lethal	
12	12	CG8055	FBgn0086656	shrb	shrb,shrub,Snf7/Vps3...	KK	106823	Lethal	
13	13	CG9012	FBgn0000319	Chc	Clathrin heavy chain	KK	108557	Lethal	
14	14	CG10882	FBgn0262126	gho	(Ghost (sec23-24)	KK	106929	Lethal	
15	15	CG13387	FBgn0020497	emb	embargoed	KK	103767	Lethal	
16	16	CG14813	FBgn0028969	deltaCOP	delta-coatomer protein	KK	109455	Lethal	
17	17	CG1913	FBgn0026103	Dser\alphaTub84B	alpha-Tubulin at 84B	KK	107109	Lethal	
18	18	CG2637	FBgn0262743	Fs(2)Ket	Female sterile (2) Ketel	KK	107622	Lethal	
19	19	CG2671	FBgn0002121	l(2)gl	lethal (2) giant larvae	KK	109604	Lethal	
20	20	CG4738	FBgn0262647	Nup160	nuclear pore protein 160	KK	109318	Lethal	
21	21	CG6251	FBgn0034118	Nup62	Nup62	KK	100588	Lethal	
22	22	CG6501	FBgn0034243	ns2	nucleostemin 2	KK	105605	Lethal	
23	23	CG9539	FBgn0086357	Sec61alpha	Sec61alpha	KK	109660	Lethal	
24	24	CG6056	FBgn0043012	AP-2sigma	sigma2,AP-2sigma,cg6...	KK	110725	Lethal	
25	25	CG2331	FBgn0261014	TER94	TRANSITIONAL ENDOPLASMIC RETICULUM ATPASE (PTHR23077:SF18)	GD	24354	Lethal	
26	26	CG8269	FBgn0021825	Dmn	DYNACTIN SUBUNIT (PTHR15346)	KK	110741	Lethal	
27	27	CG3539	FBgn0264978	Slh	SLY-1 homologous	KK	105669	Notches	
28	28	CG12276	FBgn0029512	Aos1	Sae1,SAE1,Smt3 activ...	GD	47256	Notches	
29	29	CG14542	FBgn0039402	vps2	vps2	GD	24869	Notches	
30	30	CG3161	FBgn0262736	Vha16-1	Vacuolar H+ ATPase 16kD subunit	KK	104490	Notches	
31	31	CG7007	FBgn0028662	VhaPPA1-1	VhaPPA1-1	GD	33343	Notches	
32	32	CG17332	FBgn0027779	VhaSFD	Vacuolar H+-ATPase SFD subunit	GD	47471	Notches	
33	33	CG8029	FBgn0262515	VhaAC45	CG8029	KK	101726	Notches	
34	34	CG18803	FBgn0019947	Psn	Presenilin	KK	101379	Notches	
	**35**	**CG6095**	**FBgn0260946**	**exo84**	**exo84**	**GD**	**30112**	**Notches**	
**35**	**36**	**CG3885**	**FBgn0086475**	**sec3**	**sec3**	**KK**	**108085**	**Notches**	
**36**	**37**	**CG8843**	**FBgn0031537**	**sec5**	**sec5**	**GD**	**28873**	**Notches**	
**37**	**38**	**CG5341**	**FBgn0034367**	**sec6**	**sec6**	**KK**	**105836**	**Notches**	
**38**	**39**	**CG2095**	**FBgn0262603**	**sec8**	**sec8**	**KK**	**105653**	**Notches**	
**39**	**40**	**CG7034**	**FBgn0038856**	**sec15**	**sec15**	**KK**	**105126**	**Notches**	
40	41	CG1472	FBgn0033460	sec24	sec24	KK	107154	Notches	
41	42	CG7578	FBgn0045592	Dsim\CG7578	sec71	KK	100300	Notches	
42	43	CG18112	FBgn0039702	Vps16B	Vps16B	KK	108454	Notches	
43	44	CG14804	FBgn0014411	Vps26	131F2S,anon-sts13,EG...	GD	18398	Notches	
	45	CG14804	FBgn0014411	Vps26	131F2S,anon-sts13,EG...	KK	110384	Notches	
44	46	CG5625	FBgn0034708	Vps35		GD	45570	Notches	
45	47	CG10711	FBgn0086785	Vps36	l(3)L5212,lethal (3)...	KK	107417	Notches	
	48	CG10711	FBgn0086785	Vps36	l(3)L5212,lethal (3)...	GD	16846	Notches	
46	49	CG10846	FBgn0040228	dyn-p25	dynactin-subunit-p25	KK	104352	Notches	
47	50	CG10923	FBgn0004379	Klp67A	Kinesin-like protein at 67A	KK	108852	Notches	
48	51	CG11678	FBgn0011741	Arp6	Actin-related protein 13E	KK	108081	Notches	
49	52	CG12235	FBgn0031050	Arp10	Arp11	KK	104010	Notches	
50	53	CG13089	FBgn0068450	Dyak\GE18782	(GPI transamidase subunit PIG-U)	KK	105420	Notches	
51	54	CG1404	FBgn0020255	ran	ran (small GTPase binding)	KK	104417	Notches	
52	55	CG1422	FBgn0040087	p115	p115	KK	103350	Notches	
53	56	CG2158	FBgn0033264	Nup50	NUp50	KK	100564	Notches	
54	57	CG2621	FBgn0003371	sgg	shaggy	KK	101538	Notches	
55	58	CG31229	FBgn0051229	CG31229	(some mitochondrial protein?)	KK	106764	Notches	
56	59	CG3157	FBgn0260639	gammaTub23C	gamma-Tubulin at 23C	KK	107572	Notches	
57	60	CG3248	FBgn0031536	Cog3	Cog3	KK	108574	Notches	
58	61	CG4214	FBgn0011708	Syx5	Syntaxin 5	KK	108928	Notches	
59	62	CG4579	FBgn0021761	Nup154	Nup154	KK	106136	Notches	
60	63	CG4780	FBgn0260856	membrin	-	KK	109404	Notches	
61	64	CG5808	FBgn0027617	CG5808	-	KK	103789	Notches	
62	65	CG6177	FBgn0026634	ldlCp	ldlCp-related protein	KK	103557	Notches	
63	66	CG6773	FBgn0024509	sec13	sec13	KK	110428	Notches	
64	67	CG7654	FBgn0036928	Tom20	Translocase of outer membrane 20	KK	107186	Notches	
65	68	CG8308	FBgn0087040	alphaTub67C	alpha-Tubulin at 67C	KK	108044	Notches	
66	69	CG8571	FBgn0016983	smid	smallminded	KK	108178	Notches	
67	70	CG9195	FBgn0068642	Dsim\Scamp	Scamp	KK	106761	Notches	
68	71	CG9298	FBgn0260861	Trs23	-	KK	101470	Notches	
69	72	CG9580	FBgn0067861	Sdic1	Sperm-specific dynein intermediate chain 1	KK	103054	Notches	
70	73	CG9906	FBgn0264077	Cnx14D	(Calnexin)	KK	102924	Notches	
71	74	CG1250	FBgn0262125	sec23	l(3)j13C8,sec23,Sec2...	KK	110568	Notches	
72	75	CG8487	FBgn0264560	garz	sec7-family GEF,CG 8...	GD	42140	Notches	
73	76	CG8605	FBgn0035762	CG8605	RAD50-INTERACTING PROTEIN 1 (PROTEIN RINT-1) (PTHR13520)	KK	109761	Notches	
74	77	CG6223	FBgn0085110	Dmir\CG6223	beta-coatomer protein	KK	109641	Notches	
75	78	CG4109	FBgn0036643	Syx8	syntaxin,Syntaxin,Sy...	KK	107014	Notches	
76	79	CG8266	FBgn0033339	sec31	sec31,dSec31p	GD	35867	Notches	
77	80	CG2848	FBgn0031456	Trn-SR	Transportin-Serine/A...	GD	40991	Notches	
78	81	CG12404	FBgn0032465	CG12404		KK	106143	Notches	
79	82	CG6637	FBgn0260940	lsn	larsen	KK	100908	Notches	Small notches
80	83	CG3985	FBgn0004575	Syn	Synapsin	KK	109587	Notches	Small notches
81	84	CG6025	FBgn0000115	Arf72A	Arflike at 72A	KK	106474	Notches	Small notches
	85	CG6637	FBgn0260940	lsn	larsen	KK	110350	Notches	Small notches
82	86	CG12042	FBgn0033206	CG12042	P62 DYNACTIN	GD	31623	Notches	Small Notches
83	87	CG9033	FBgn0033629	Tsp47F	Dm.Tsp47F,Tetraspani...	GD	44288	Notches	Small Notches
84	88	CG12839	FBgn0033135	Tsp42En	BcDNA:RH02160,Dm.Tsp...	GD	37474	Notches	Small Notches
85	89	CG12838	FBgn0033136	Tsp42Eo	BcDNA:RH51444,Dm.Tsp...	KK	103222	Notches	Small Notches
86	90	CG1088	FBgn0015324	Vha26	Vacuolar H+-ATPase 26kD E subunit	KK	102378	Notches	Small Notches
87	91	CG8048	FBgn0262511	Vha44	Vacuolar H+ ATPase 44kD C subunit	KK	101527	Notches	Small Notches
	92	CG5625	FBgn0034708	Vps35		GD	22180	Notches	Small Notches
88	93	CG10198	FBgn0039120	Nup98-96	Nup98	KK	109279	Notches	Small Notches
89	94	CG1098	FBgn0027497	Madm	MLF1-adaptor molecule	KK	101758	Notches	Small Notches
90	95	CG1099	FBgn0023388	Dap160	Dynamin associated protein 160	KK	106689	Notches	Small Notches
91	96	CG1158	FBgn0037310	Tim17b1	Tim17b1	KK	103529	Notches	Small Notches
92	97	CG12752	FBgn0028411	Nxt1	NTF2-related export protein 1	KK	103146	Notches	Small Notches
93	98	CG1359	FBgn0260860	Bet5	-	KK	108748	Notches	Small Notches
94	99	CG13692	FBgn0031254	CG13692	Arf-like activity?	KK	102036	Notches	Small Notches
95	100	CG3139	FBgn0004242	Syt1	Synaptotagmin 1	KK	100608	Notches	Small Notches
96	101	CG32683	FBgn0052683	CG32683	(arrestin like)	KK	104029	Notches	Small Notches
97	102	CG32697	FBgn0028341	l(1)G0232	lethal (1) G0232	KK	104427	Notches	Small Notches
98	103	CG4869	FBgn0003890	betaTub97EF	beta-Tubulin at 97EF	KK	105075	Notches	Small Notches
99	104	CG5014	FBgn0029687	Vap-33-1	Vap-33-1	KK	100809	Notches	Small Notches
100	105	CG6948	FBgn0024814	Clc	Clathrin light chain	KK	106632	Notches	Small Notches
101	106	CG7051	FBgn0263988	Dic61B	-	KK	101248	Notches	Small Notches
102	107	CG7293	FBgn0004381	Klp68D	Kinesin-like protein at 68D	KK	101058	Notches	Small Notches
103	108	CG7359	FBgn0260855	Sec22	-	KK	100766	Notches	Small Notches
104	109	CG7398	FBgn0024921	Trn	Transportin	KK	105181	Notches	Small Notches
105	110	CG8722	FBgn0033247	Nup44A	Nup44A	KK	106489	Notches	Small Notches
106	111	CG8831	FBgn0033737	Nup54	-	KK	103724	Notches	Small Notches
107	112	CG9474	FBgn0028401	Snap24	Synapse protein 24	KK	108209	Notches	Small Notches
108	113	CG9476	FBgn0003886	alphaTub85E	alpha-Tubulin at 85E	KK	103202	Notches	Small Notches
109	114	CG3511	FBgn0035027	CG3511	-	KK	110015	Notches	Small Notches
110	115	CG12298	FBgn0003545	sub	KINESIN FAMILY MEMBER 20 (RABKINESIN-6) (PTHR16012:SF61)	GD	45402	Notches	Small Notches
111	116	CG31048	FBgn0264324	spg	DOCK-3,4 (PTHR23317:SF28)	GD	21293	Notches	Small Notches
	117	CG8266	FBgn0033339	sec31	sec31,dSec31p	GD	35868	Notches	Small Notches
112	118	CG11278	FBgn0036341	Syx13	l(3)01470,syntaxin,S...	KK	102432	Notches	Small Notches

113	119	CG1708	FBgn0000352	cos	costa	KK	108914	No phenotype (remarks)	Other defects
114	120	CG8479	FBgn0261276	opa1-like	optic atrophy 1-like	KK	106290	No phenotype (remarks)	Other defects
115	121	CG7558	FBgn0262716	Arp3	Actin-related protein 66B	KK	108951	No phenotype (remarks)	Other defects
	122	CG17515	FBgn0039966	Rab21	Rab21	GD	32941	No phenotype (remarks)	Other defects
116	123	CG12403	FBgn0020368	Vha68-1	Vha68-1	KK	108701	No phenotype	
117	124	CG3269	FBgn0014009	Rab2	Rab-protein 2	KK	105358	No phenotype	
118	125	CG7576	FBgn0005586	Rab3	Rab-protein 3	KK	100787	No phenotype	
119	126	CG4921	FBgn0016701	Rab4	Rab-protein 4	KK	106651	No phenotype	
	127	CG4921	FBgn0016701	Rab4	Rab-protein 4	GD	24672	No phenotype	
120	128	CG5915	FBgn0015795	Rab7	Rab-protein 7	GD	40338	No phenotype	
121	129	CG9994	FBgn0032782	Rab9	Rab9	KK	107192	No phenotype	
	130	CG9994	FBgn0032782	Rab9	Rab9	GD	36200	No phenotype	
	131	CG9994	FBgn0032782	Rab9	Rab9	GD	36201	No phenotype	
	132	CG9994	FBgn0032782	Rab9	Rab9	GD	43486	No phenotype	
122	133	CG32678	FBgn0067052	Rab9D	Rab GTPase 9D	KK	109376	No phenotype	
	134	CG32678	FBgn0067052	Rab9D	Rab GTPase 9D	GD	49738	No phenotype	
123	135	CG9807	FBgn0030221	Rab9Db	Rab GTPase 9Db	KK	109220	No phenotype	
	136	CG9807	FBgn0030221	Rab9Db	Rab GTPase 9Db	KK	109089	No phenotype	
	137	CG9807	FBgn0030221	Rab9Db	Rab GTPase 9Db	GD	29282	No phenotype	
124	138	CG32671	FBgn0052671	Rab9Fa	Rab GTPase 9Fa	KK	116172	No phenotype	
	139	CG17515	FBgn0039966	Rab21	Rab21	KK	109991	No phenotype	
125	140	CG34410	FBgn0086913	Rab26	Rab26	KK	101330	No phenotype	
	141	CG34410	FBgn0086913	Rab26	Rab26	GD	43730	No phenotype	
126	142	CG8024	FBgn0002567	ltd	Rab32	KK	104348	No phenotype	
127	143	CG12156	FBgn0029959	Rab39	Rab39	GD	31665	No phenotype	
128	144	CG1900	FBgn0030391	Rab40	Rab40	KK	110563	No phenotype	
129	145	CG3870	FBgn0015372	RabX1	chrowded	KK	103039	No phenotype	
130	146	CG2885	FBgn0030200	RabX2	RabX2	KK	103311	No phenotype	
	147	CG2885	FBgn0030200	RabX2	RabX2	GD	49851	No phenotype	
131	148	CG32670	FBgn0052670	Rab9Fb	Rab GTPase 9Fb	KK	102312	No phenotype	
132	149	CG7980	FBgn0035255	RabX5	RabX5	KK	103630	No phenotype	
	150	CG7980	FBgn0035255	RabX5	RabX5	GD	28169	No phenotype	
133	151	CG12015	FBgn0035155	RabX6	RabX6	KK	101654	No phenotype	
	152	CG12015	FBgn0035155	RabX6	RabX6	GD	50817	No phenotype	
134	153	CG7062	FBgn0015793	Rab19	Rab-related protein 3	KK	103653	No phenotype	
	154	CG7062	FBgn0015793	Rab19	Rab-related protein 3	GD	34190	No phenotype	
135	155	CG6213	FBgn0026753	Vha13	Vacuolar H+ ATPase G-subunit	KK	106536	No phenotype	
136	156	CG1076	FBgn0037402	Vha14-2		KK	102478	No phenotype	
137	157	CG32089	FBgn0028668	Vha16-2	Vha16-2	KK	106520	No phenotype	
138	158	CG32090	FBgn0028667	Vha16-3	Vha16-3	KK	102067	No phenotype	
139	159	CG9013	FBgn0262513	Vha16-4	Vha16-4	KK	107375	No phenotype	
140	160	CG6737	FBgn0032294	Vha16-5	Vha16-5	KK	107481	No phenotype	
141	161	CG8186/CG7433	unknown ID		Vha36	KK	110468	No phenotype	
142	162	CG13167	FBgn0033706	Vha36-2	CG13167	KK	104111	No phenotype	
143	163	CG8310	FBgn0040377	Vha36-3	CG8310	KK	104048	No phenotype	
144	164	CG5075	FBgn0032464	Vha68-3	Vacuolar H+-ATPase 68kD A subunit	GD	41646	No phenotype	
145	165	CG1709	FBgn0028671	Vha100-1	Vha100-1	KK	108905	No phenotype	
146	166	CG30329	FBgn0028669	Vha100-3	Vha100-3	KK	110051	No phenotype	
147	167	CG12602	FBgn0032373	Vha100-5	CG12602	KK	106611	No phenotype	
148	168	CG4624	FBgn0039058	VhaAC39-2		GD	34303	No phenotype	
149	169	CG11589	FBgn0028664	VhaM9.7-c	VhaM9.7-1	KK	101574	No phenotype	
150	170	CG7625	FBgn0028663	VhaM9.7-b	VhaM9.7-2	GD	30384	No phenotype	
151	171	CG14909	FBgn0038458	VhaM9.7-d		KK	106115	No phenotype	
152	172	CG1268	FBgn0035521	VhaM9.7-a	CG1268	KK	104315	No phenotype	
153	173	CG7026	FBgn0262514	VhaPPA1-2	VhaPPA1-2	GD	48830	No phenotype	
154	174	CG13762	FBgn0040333	brv3	CG13762	KK	101019	No phenotype	
**155**	**175**	**CG7127**	**FBgn0035892**	**exo70**	**exo70**	**KK**	**103717**	**No phenotype**	
156	176	CG14214	FBgn0031049	Sec61gamma	Sec61gamma	KK	100603	No phenotype	
157	177	CG8454	FBgn0261241	Vps16A	dVps16A,Vps16A	GD	23769	No phenotype	
	178	CG4071	FBgn0034744	Vps20	anon-EST:Posey40,vps...	GD	26388	No phenotype	
158	179	CG3093	FBgn0000482	dor	deep orange	KK	102176	No phenotype	
159	180	CG5373	FBgn0015277	Pi3K59F	Phosphotidylinositol 3 kinase 59F	KK	107602	No phenotype	
160	181	CG4471	FBgn0033137	Tsp42Ep	Tetraspanin 42Ep	KK	108515	No phenotype	
161	182	CG9494	FBgn0032074	Tsp29Fa	Tetraspanin 29Fa	KK	105839	No phenotype	
162	183	CG9093	FBgn0031760	Tsp26A	Tetraspanin 26A	KK	101473	No phenotype	
163	184	CG8666	FBgn0032943	Tsp39D	Tetraspanin 39D	KK	107776	No phenotype	
164	185	CG4999	FBgn0035936	Tsp66E	Tetraspanin 66E	KK	104430	No phenotype	
165	186	CG4690	FBgn0029837	Tsp5D	Dm.Tsp5D,Tetraspanin...	GD	45740	No phenotype	
166	187	CG32136	FBgn0043550	Tsp68C	Dm.Tsp68C,Tetraspani...	GD	37441	No phenotype	
167	188	CG18817	FBgn0029508	Tsp42Ea	Q9U3V4,BcDNA:GH05668...	KK	109172	No phenotype	
168	189	CG18816	FBgn0042086	Tsp42Eb	LP06288,BEST:LP06288...	GD	49692	No phenotype	
169	190	CG14936	FBgn0032376	Tsp33B	Tetraspanin 33B	KK	104079	No phenotype	
	191	CG14936	FBgn0032376	Tsp33B	Dm.Tsp33B,Tetraspani...	GD	6123	No phenotype	
170	192	CG14468	FBgn0033042	Tsp42A	Dm.Tsp42A,Tetraspani...	KK	109889	No phenotype	
171	193	CG12847	FBgn0033124	Tsp42Ec	Dm.Tsp42Ec,tetraspan...	GD	37149	No phenotype	
	194	CG12847	FBgn0033124	Tsp42Ec	Dm.Tsp42Ec,tetraspan...	GD	43534	No phenotype	
172	195	CG12844	FBgn0033129	Tsp42Eh	Dm.Tsp42Eh,tetraspan...	KK	102178	No phenotype	
	196	CG12844	FBgn0033129	Tsp42Eh	Dm.Tsp42Eh,tetraspan...	GD	48973	No phenotype	
173	197	CG12843	FBgn0033130	Tsp42Ei	Tetraspanin 42Ei	KK	101044	No phenotype	
	198	CG12843	FBgn0033130	Tsp42Ei	Dm.Tsp42Ei,tetraspan...	KK	101044	No phenotype	
174	199	CG12841	FBgn0033133	Tsp42Ek	Tetraspanin 42Ek	KK	102294	No phenotype	
	200	CG12839	FBgn0033135	Tsp42En	BcDNA:RH02160,Dm.Tsp...	GD	37473	No phenotype	
175	201	CG12837	FBgn0033139	Tsp42Er	Dm.Tsp42Er,tetraspan...	KK	104950	No phenotype	
176	202	CG12832	FBgn0033138	Tsp42Eq	Tetraspanin 42Eq	KK	101926	No phenotype	
177	203	CG12143	FBgn0033132	Tsp42Ej	Dm.Tsp42Ej,sun,Sun,s...	KK	105191	No phenotype	
178	204	CG12142	FBgn0033128	Tsp42Eg	BcDNA:GM06962,Dm.Tsp...	KK	108430	No phenotype	
	205	CG32136	FBgn0043550	Tsp68C	Tetraspanin 68C	KK	101945	No phenotype	
179	206	CG10067	FBgn0000044	Act57B	Actin 57B	KK	102129	No phenotype	
180	207	CG10090	FBgn0038018	Tim17a1	Tim17a1	KK	101139	No phenotype	
181	208	CG10188	FBgn0032796	CG10188	-	KK	103391	No phenotype	
182	209	CG10225	FBgn0039110	RanBP3	-	KK	104432	No phenotype	
183	210	CG10478	FBgn0035657	alphaKap4	-	KK	108143	No phenotype	
184	211	CG10642	FBgn0004380	Klp64D	Kinesin-like protein at 64D	KK	103358	No phenotype	
185	212	CG10718	FBgn0004374	neb	nebbish	KK	108138	No phenotype	
186	213	CG10907	FBgn0036207	CG10907	-	KK	103623	No phenotype	
187	214	CG11092	FBgn0045656	Dsim\CG11092	-	KK	100315	No phenotype	
188	215	CG11110	FBgn0034535	CG11110	-	KK	106768	No phenotype	
189	216	CG11173	FBgn0034913	usnp	ubisnap	KK	107947	No phenotype	
190	217	CG11211	FBgn0033067	CG11211	-	KK	107118	No phenotype	
191	218	CG11611	FBgn0036204	Tim13	Tim13	KK	100776	No phenotype	
192	219	CG11759	FBgn0028421	Kap3	Kinesin associated protein 3	KK	103548	No phenotype	
193	220	CG11771	FBgn0039252	CG11771	-	KK	108188	No phenotype	
194	221	CG11838	FBgn0260933	rempA	reduced mechanoreceptor potential A	KK	103424	No phenotype	
195	222	CG11958	FBgn0015622	Cnx99A	Calnexin 99A	KK	100740	No phenotype	
196	223	CG12051	FBgn0000043	Act42A	Actin 42A	KK	104731	No phenotype	
197	224	CG12092	FBgn0261675	Npc1b	NPC1b	KK	108054	No phenotype	
198	225	CG12147	FBgn0037325	CG12147	-	KK	101875	No phenotype	
199	226	CG12500	FBgn0016976	stnA	stoned A	KK	105203	No phenotype	
200	227	CG12817	FBgn0037798	CG12817	-	KK	106952	No phenotype	
201	228	CG13137	FBgn0032188	CG13137	-	KK	107865	No phenotype	
202	229	CG13396	FBgn0001084	fy	fuzzy	KK	108550	No phenotype	
203	230	CG13425	FBgn0015907	bl	bancal	KK	105271	No phenotype	
204	231	CG13887	FBgn0035165	CG13887	-	KK	106452	No phenotype	
205	232	CG13892	FBgn0035141	Cypl	Cyclophilin-like	KK	104316	No phenotype	
206	233	CG13930	FBgn0035256	CG13930	-	KK	104545	No phenotype	
207	234	CG1418	FBgn0033468	CG1418	-	KK	104571	No phenotype	
208	235	CG14411	FBgn0084085	Dyak\GE17154	-	KK	109622	No phenotype	
209	236	CG14535	FBgn0031955	CG14535	-	KK	108308	No phenotype	
210	237	CG14666	FBgn0037307	Tim17a2	Tim17a2	KK	107739	No phenotype	
211	238	CG1467	FBgn0031106	Syx16	Syntaxin 16	KK	109504	No phenotype	
212	239	CG14690	FBgn0037828	tomboy20	tomboy20	KK	100931	No phenotype	
213	240	CG14838	FBgn0035799	CG14838	-	KK	105707	No phenotype	
214	241	CG1487	FBgn0040206	krz	kurtz	KK	103756	No phenotype	
215	242	CG1514	FBgn0068694	Dsim\GD16870	-	KK	105671	No phenotype	
216	243	CG15701	FBgn0034095	CG15701	os	KK	107983	No phenotype	

217	244	CG15844	FBgn0263029	CG43324	Kinesin-like protein at 54D	KK	100140	No phenotype	
218	245	CG1599	FBgn0033452	CG1599	-	KK	108733	No phenotype	
219	246	CG1618	FBgn0000346	comt	comatose	KK	105552	No phenotype	
220	247	CG1657	FBgn0030286	CG1657	-	KK	108453	No phenotype	
221	248	CG1660	FBgn0030480	Tim9a	Tim9a	KK	104476	No phenotype	
222	249	CG16834	FBgn0040096	lectin-33A	lectin-33A	KK	108412	No phenotype	
223	250	CG16892	FBgn0030122	CG16892	-	KK	101415	No phenotype	
224	251	CG16932	FBgn0035060	Eps-15	EGF receptor pathway substrate clone 15	KK	106678	No phenotype	
225	252	CG17262	FBgn0243513	cnir	cornichon related	KK	104009	No phenotype	
226	253	CG17271	FBgn0038829	CG17271	-	KK	101754	No phenotype	
	254	CG17271	FBgn0038829	CG17271	-	KK	103101	No phenotype	
227	255	CG17566	FBgn0010097	gammaTub37C	gamma-Tubulin at 37C	KK	109921	No phenotype	
228	256	CG18000	FBgn0003654	sw	short wing	KK	101559	No phenotype	
229	257	CG18290	FBgn0000046	Act87E	Actin 87E	KK	102480	No phenotype	
230	258	CG1924	FBgn0030377	CG1924	-	KK	102773	No phenotype	
231	259	CG2219	FBgn0039889	CG2219	-	KK	107995	No phenotype	
232	260	CG2520	FBgn0083813	Dbuz\CG2520	like-AP180	KK	105767	No phenotype	
233	261	CG2577	FBgn0030384	CG2577	-	KK	105471	No phenotype	
234	262	CG2715	FBgn0024980	Syx4	Syntaxin 4	KK	102466	No phenotype	
235	263	CG2852	FBgn0034753	CG2852	-	KK	102376	No phenotype	
236	264	CG2999	FBgn0025726	unc-13	unc-13	KK	101383	No phenotype	
	265	CG3093	FBgn0000482	dor	deep orange	KK	107053	No phenotype	
237	266	CG31495	FBgn0051495	CG31495	-	KK	105777	No phenotype	
238	267	CG31721	FBgn0051721	Trim9	Trim9	KK	100767	No phenotype	
239	268	CG31787	FBgn0051787	CG31787	-	KK	107111	No phenotype	
240	269	CG32239	FBgn0084660	Dmir\CG32239	Guanine nucleotide exchange factor GEF64C	KK	105252	No phenotype	
241	270	CG32350	FBgn0052350	CG32350	-	KK	107420	No phenotype	
242	271	CG32381	FBgn0035756	unc-13-4A	unc-13-4A	KK	109304	No phenotype	
243	272	CG32392	FBgn0052392	CG32392	-	KK	100592	No phenotype	
244	273	CG32562	FBgn0028974	xmas-2	xmas-2	KK	104156	No phenotype	
245	274	CG32701	FBgn0028327	l(1)G0320	lethal (1) G0320	KK	110344	No phenotype	
246	275	CG33180	FBgn0053180	Ranbp16	Ranbp16	KK	107391	No phenotype	
247	276	CG33214	FBgn0264561	Glg1	-	KK	108284	No phenotype	
248	277	CG3401	FBgn0003888	betaTub60D	beta-Tubulin at 60D	KK	102052	No phenotype	
	278	CG3401	FBgn0003888	betaTub60D	beta-Tubulin at 60D	KK	104937	No phenotype	
249	279	CG34397	FBgn0085426	Rgk3	Rgk3	KK	106428	No phenotype	
250	280	CG34414	FBgn0085443	spri	sprint	KK	101164	No phenotype	
251	281	CG3499	FBgn0034792	CG3499	-	KK	105143	No phenotype	
252	282	CG3585	FBgn0023458	Rbcn-3A	Rabconnectin-3A	KK	108547	No phenotype	
253	283	CG3869	FBgn0029870	Marf	Mitochondrial assembly regulatory factor	KK	105261	No phenotype	
254	284	CG3911	FBgn0260859	Bet3	-	KK	104469	No phenotype	
255	285	CG3988	FBgn0028552	gammaSnap	gamma-soluble NSF attachment protein	KK	100738	No phenotype	
256	286	CG4030	FBgn0034585	CG4030	-	KK	110346	No phenotype	
257	287	CG4074	FBgn0037017	CG4074	-	KK	104859	No phenotype	
258	288	CG4325	FBgn0026878	CG4325	-	KK	102993	No phenotype	
259	289	CG4453	FBgn0086002	Dsim\Nup153	Nup153	KK	107750	No phenotype	
260	290	CG4532	FBgn0029903	pod1	pod1	KK	108886	No phenotype	
261	291	CG4663	FBgn0033812	Pex13	-	KK	108829	No phenotype	
262	292	CG4764	FBgn0084168	Dsim\GD23074	-	KK	101375	No phenotype	
263	293	CG4789	FBgn0030792	CG4789	-	KK	101535	No phenotype	
264	294	CG4799	FBgn0011823	Pen	Pendulin	KK	102627	No phenotype	
265	295	CG4886	FBgn0028382	cyp33	cyclophilin-33	KK	108734	No phenotype	
266	296	CG5020	FBgn0020503	CLIP-190	Cytoplasmic linker protein 190	KK	107176	No phenotype	
267	297	CG5026	FBgn0035945	CG5026	-	KK	105674	No phenotype	
268	298	CG5071	FBgn0039347	CG5071	-	KK	101932	No phenotype	
269	299	CG5140	FBgn0034314	nopo	no poles	KK	104477	No phenotype	
270	300	CG5373	FBgn0015277	Pi3K59F	Phosphotidylinositol 3 kinase 59F	KK	100296	No phenotype	
271	301	CG5409	FBgn0011743	Arp53D	Actin-related protein 53D	KK	108369	No phenotype	
272	302	CG5439	FBgn0032476	CG5439	-	KK	109602	No phenotype	
273	303	CG5498	FBgn0027565	CG5498	-	KK	103711	No phenotype	
274	304	CG5559	FBgn0261089	Sytalpha	Synaptotagmin alpha	KK	100957	No phenotype	
275	305	CG5662	FBgn0030620	CG5662	-	KK	102645	No phenotype	
276	306	CG5722	FBgn0024320	Npc1a	Niemann-Pick type C-1a	KK	105405	No phenotype	
277	307	CG5734	FBgn0032191	CG5734	-	KK	109452	No phenotype	
278	308	CG5885	FBgn0068285	Dyak\GE10193	-	KK	105974	No phenotype	
279	309	CG5886	FBgn0039379	CG5886	-	KK	106471	No phenotype	
280	310	CG6053	FBgn0036195	CG6053	-	KK	108601	No phenotype	
281	311	CG6129	FBgn0039152	Rootletin	-	KK	110171	No phenotype	
282	312	CG6196	FBgn0038323	CG6196	-	KK	101556	No phenotype	
283	313	CG6202	FBgn0019925	Surf4	Surfeit 4	KK	108944	No phenotype	
284	314	CG6208	FBgn0037789	CG6208	-	KK	105575	No phenotype	
285	315	CG6259	FBgn0036740	CG6259	-	KK	101422	No phenotype	
286	316	CG6395	FBgn0004179	Csp	Cysteine string protein	KK	103201	No phenotype	
287	317	CG6486	FBgn0035922	Pex7	-	KK	108795	No phenotype	
288	318	CG6512	FBgn0036702	CG6512	-	KK	109629	No phenotype	
289	319	CG6527	FBgn0036085	CG6527	-	KK	100865	No phenotype	
290	320	CG6549	FBgn0024689	fws	four way stop	KK	110378	No phenotype	
291	321	CG6560	FBgn0038916	dnd	dead end	KK	104311	No phenotype	
292	322	CG6822	FBgn0035909	ergic53	ergic53	KK	108445	No phenotype	
293	323	CG7094	FBgn0032650	CG7094	-	KK	108273	No phenotype	
294	324	CG7371	FBgn0031710	CG7371	-	KK	106958	No phenotype	
295	325	CG7452	FBgn0035540	Syx17	Syntaxin 17	KK	108825	No phenotype	
296	326	CG7609	FBgn0027518	CG7609	-	KK	108562	No phenotype	
297	327	CG7700	FBgn0044871	Gos28	Gos28	KK	100289	No phenotype	
298	328	CG7735	FBgn0034446	CG7735	-	KK	104462	No phenotype	
299	329	CG7736	FBgn0037084	Syx6	Syntaxin 6	KK	104795	No phenotype	
300	330	CG7747	FBgn0034109	CG7747	-	KK	105644	No phenotype	
301	331	CG7787	FBgn0032020	CG7787	-	KK	105730	No phenotype	
302	332	CG7791	FBgn0033038	CG7791	-	KK	109658	No phenotype	
303	333	CG7794	FBgn0038565	CG7794	-	KK	104265	No phenotype	
304	334	CG7846	FBgn0030877	Arp8	Actin-related protein 8	KK	104425	No phenotype	
305	335	CG7864	FBgn0035233	Pex10	-	KK	110405	No phenotype	
306	336	CG8156	FBgn0013750	Arf51F	ADP ribosylation factor 51F	KK	100726	No phenotype	
307	337	CG8183	FBgn0019968	Khc-73	Kinesin-73	KK	105984	No phenotype	
308	338	CG8219	FBgn0035693	CG8219	-	KK	103487	No phenotype	
309	339	CG8226	FBgn0033357	Tom7	Translocase of outer membrane 7	KK	102728	No phenotype	
310	340	CG8274	FBgn0013756	Mtor	Megator	KK	110218	No phenotype	
311	341	CG8315	FBgn0034058	Pex11	-	KK	105654	No phenotype	
312	342	CG8330	FBgn0033074	tomboy40	tomboy40	KK	105557	No phenotype	
313	343	CG8407	FBgn0033687	CG8407	-	KK	100696	No phenotype	
314	344	CG8548	FBgn0024889	Kap-alpha1	karyopherin alpha1	KK	108741	No phenotype	
315	345	CG8552	FBgn0261164	Dsim\CG8552	-	KK	108121	No phenotype	
316	346	CG9139	FBgn0262937	Rabex-5	-	KK	105534	No phenotype	
317	347	CG9240	FBgn0030669	CG9240	-	KK	103596	No phenotype	
318	348	CG9246	FBgn0032925	CG9246	-	KK	104868	No phenotype	
319	349	CG9279	FBgn0085123	Dmir\CG9279	-	KK	105109	No phenotype	
320	350	CG9313	FBgn0086527	Dmir\CG9313	-	KK	106167	No phenotype	
321	351	CG9359	FBgn0003889	betaTub85D	beta-Tubulin at 85D	KK	109590	No phenotype	
322	352	CG9393	FBgn0037710	CG9393	-	KK	110385	No phenotype	
323	353	CG9423	FBgn0027338	Kap-alpha3	karyopherin alpha3	KK	106249	No phenotype	
324	354	CG9446	FBgn0033109	coro	coro	KK	109644	No phenotype	
325	355	CG9543	FBgn0068192	Dyak\GE13901	epsilonCOP	KK	107588	No phenotype	
326	356	CG9901	FBgn0011742	Arp2	Actin-related protein 14D	KK	101999	No phenotype	
327	357	CG9913	FBgn0038205	Kif19A	Kif19A	KK	106569	No phenotype	
328	358	CG9962	FBgn0031441	CG9962	-	KK	108721	No phenotype	
329	359	CG8228	FBgn0261049	Vps45	Vacuolar protein sorting 45	KK	110660	No phenotype	
330	360	CG3210	FBgn0026479	Drp1	Dynamin related protein 1	GD	44156	No phenotype	
331	361	CG10653	FBgn0001202	hk	hk,hook,Hook,dHk	GD	35483	No phenotype	
332	362	CG4027	FBgn0000042	Act5C	l(1)G0009,l(1)G0010...	KK	101438	No phenotype	
333	363	CG10950	FBgn0034205	CG10950	TRANSPORTIN 3 (PTHR12363:SF5)	GD	41462	No phenotype	
334	364	CG11857	FBgn0039303	CG11857	RER1 PROTEIN (PTHR10743)	GD	23204	No phenotype	
335	365	CG3279	FBgn0260862	Vti1	VESICLE TRANSPORT V-SNARE PROTEIN VTI1A (PTHR21230:SF3)	KK	109819	No phenotype	
336	366	CG6192	FBgn0032341	Reps	PARTNER OF RALBP-1 (PTHR11216:SF33)	KK	110704	No phenotype	
337	367	CG4289	FBgn0037020	Pex14	PEROXISOMAL MEMBRANE PROTEIN PEX14 (PTHR23058)	GD	42590	No phenotype	
338	368	CG33101	FBgn0013998	Nsf2	NEM-sensitive fusion...	GD	7743	No phenotype	

339	369	CG1763	FBgn0002948	nod	no distributive disjunction	GD	48150	No phenotype	
340	370	CG17461	FBgn0039925	Kif3C	KIF24,Kif3C,KIF3C,CG...	GD	43641	No phenotype	
341	371	CG2086	FBgn0027594	drpr	draper	GD	27086	No phenotype	
342	372	CG3530	FBgn0028497	CG3530	MYOTUBULARIN-like,Bc...	KK	110786	No phenotype	
343	373	CG10047	FBgn0028400	Syt4	synaptotagmin IV,Syn...	GD	33317	No phenotype	
344	374	CG6760	FBgn0013563	Pex1	PEROXISOME BIOGENESIS FACTOR 1 (PEROXIN-1) (PTHR23077:SF12)	GD	27743	No phenotype	
345	375	CG3966	FBgn0002936	ninaA	neither inactivation...	GD	51855	No phenotype	
346	376	CG6521	FBgn0027363	Stam	SIGNAL TRANSDUCING ADAPTER MOLECULE (STAM) (PTHR13856:SF23)	GD	22497	No phenotype	
347	377	CG10617	FBgn0261085	Syt12	SYNAPTOTAGMIN-12 (PTHR10024:SF15)	KK	110655	No phenotype	
348	378	CG7197	FBgn0085149	Dsim\GD13004	ADP-RIBOSYLATION FACTOR-LIKE 5, ARL5 (PTHR11711:SF31)	GD	19736	No phenotype	
349	379	CG1724	FBgn0031164	CG1724	BcDNA:AT13347	GD	30317	No phenotype	
350	380	CG7765	FBgn0001308	Khc	khc,Khc,KHC,KIF5A,ki...	GD	44337	No phenotype	
351	381	CG6392	FBgn0040232	cmet	KINESIN-LIKE KINETOCHORE MOTOR PROTEIN CENP (PTHR16012:SF190)	GD	35081	No phenotype	
352	382	CG3639	FBgn0031282	Pex12	PEROXISOME ASSEMBLY PROTEIN 12 (PEROXIN-12) (PTHR12888)	GD	34671	No phenotype	
353	383	CG9878	FBgn0027360	Tim10	MITOCHONDRIAL INNER MEMBRANE TRANSLOCASE SUBUNIT TIM10 (PTHR11038:SF8	KK	107111	No phenotype	
354	384	CG32593	FBgn0264078	Flo-2	CG11547,CG14409,Flo-...	GD	31525	No phenotype	
355	385	CG11856	FBgn0039302	Nup358	Nup358	GD	38583	No phenotype	
356	386	CG9308	FBgn0034681	CG9308	COPII-COATED VESICLE MEMBRANE PROTEIN P24 (PTHR22811:SF11)	GD	6606	No phenotype	
357	387	CG9778	FBgn0261086	Syt14	Synaptotagmin 14	GD	11037	No phenotype	
358	388	CG10986	FBgn0082798	Dpai\g	garnet APL5 PROTEIN (PTHR22781:SF9)	GD	41369	No phenotype	
359	389	CG3002	FBgn0030141	Gga	dmGGA,Gga,GGA	GD	3270	No phenotype	
360	390	CG1333	FBgn0261274	Ero1L	kiga,Endoplasmic ret...	KK	110454	No phenotype	
361	391	CG5510	FBgn0039160	CG5510	VESICULAR INTEGRAL-MEMBRANE PROTEIN VIP36 (PTHR12223:SF6)	KK	109494	No phenotype	
362	392	CG7225	FBgn0004003	wbl	wbl,wbt,wind,Wind,Wi...	GD	13864	No phenotype	
363	393	CG5521	FBgn0039466	CG5521	TUBERIN (PTHR10063)	KK	110722	No phenotype	
364	394	CG8566	FBgn0034155	unc-104	imac,KIF1B,Klp53D,un...	GD	23465	No phenotype	
365	395	CG33162	FBgn0011509	SrpRbeta	Signal recognition particle receptor beta	KK	110760	No phenotype	
366	396	CG10859	FBgn0032520	CG10859		GD	27322	No phenotype	
367	397	CG2381	FBgn0039900	Syt7	Synaptotagmin VII,Sy...	GD	24988	No phenotype	
368	398	CG9212	FBgn0030724	Nipsnap	Nipsnap	GD	28115	No phenotype	
369	399	CG5127	FBgn0039335	Vps33B	Vacuolar protein sorting 33B	GD	47030	No phenotype	
370	400	CG4420	FBgn0068678	Dsim\CG4420		GD	40512	No phenotype	
371	401	CG33555	FBgn0264754	btsz	l(3)10418,BcDNA:GH06...	KK	102608	No phenotype	
372	402	CG5962	FBgn0000121	Arr2	phosrestin-1,phosres...	GD	20991	No phenotype	
373	403	CG3652	FBgn0031600	CG3652		GD	35575	No phenotype	
374	404	CG5675	FBgn0026313	X11L	Mint,MINT,X11,X11L,X...	GD	27479	No phenotype	
375	405	CG1409	FBgn0029964	CG1409	BcDNA:AT29287,D.M.BL...	KK	110447	No phenotype	
376	406	CG16976	FBgn0264606	CG43955		KK	110099	No phenotype	
377	407	CG5433	FBgn0010235	Klc	kinesin,kinesin I,ki...	GD	39583	No phenotype	
378	408	CG11628	FBgn0086779	step	Grp1,GRP1/cytohesin ...	KK	109449	No phenotype	
379	409	CG12234	FBgn0084564	Dyak\GE17366	Ranbp21,RanBP21,dmEx...	GD	31707	No phenotype	
380	410	CG11642	FBgn0040340	TRAM	TRAM,CG18830,EG:BACR...	GD	39352	No phenotype	
381	411	CG7212	FBgn0261532	cdm	importin 13,cadmus,c...	GD	40436	No phenotype	
382	412	CG6939	FBgn0025802	Sbf	sbf,Sbf,SBF,Sbf1,SET...	GD	22317	No phenotype	
383	413	CG7815	FBgn0036497	ran-like	ran-like,Ran-like,an...	KK	109695	No phenotype	
384	414	CG14955	FBgn0035399	CG14955		KK	109712	No phenotype	
385	415	CG3632	FBgn0030735	CG3632	MYOTUBULARIN-like	KK	110167	No phenotype	
386	416	CG4520	FBgn0038355	CG4520		GD	34863	No phenotype	
387	417	CG10379	FBgn0015513	mbc	mbc,Mbc,MBC,myoblast...	GD	16044	No phenotype	
388	418	CG11427	FBgn0003210	rb	rb,ruby,AP-3beta,bet...	GD	38504	No phenotype	
389	419	CG6562	FBgn0034691	synj	IPP,synaptojanin,syn...	GD	46070	No phenotype	
390	420	CG17762	FBgn0030412	tomosyn	tomosyn,Tomosyn,BcDN...	GD	43629	No phenotype	
391	421	CG7364	FBgn0028541	TM9SF4	TM9SF4	GD	7706	No phenotype	
392	422	CG6699	FBgn0025724	beta'Cop	beta'-coatomer protein	GD	42071	No phenotype	
393	423	CG1571	FBgn0029993	CG1571	AXONEMAL DYNEIN INTERMEDIATE CHAIN (PTHR12442:SF7)	GD	51846	No phenotype	
394	424	CG3267	FBgn0042083	CG3267	l(2)4524,X11B,X11Lbe...	KK	105961	No phenotype	
